# Production and characterization of a chimeric antigen, based on nucleocapsid of SARS-CoV-2 fused to the extracellular domain of human CD154 in HEK-293 cells as a vaccine candidate against COVID-19

**DOI:** 10.1371/journal.pone.0288006

**Published:** 2023-09-26

**Authors:** Thailin Lao, Ileanet Avalos, Elsa María Rodríguez, Yasser Zamora, Alianet Rodriguez, Ailyn Ramón, Yanitza Alvarez, Ania Cabrales, Ivan Andújar, Luis Javier González, Pedro Puente, Cristina García, Leonardo Gómez, Rodolfo Valdés, Mario Pablo Estrada, Yamila Carpio

**Affiliations:** 1 Center for Genetic Engineering and Biotechnology, Animal Biotechnology Department, Havana, Cuba; 2 Center for Genetic Engineering and Biotechnology, Laboratory of Molecular Oncology, Havana, Cuba; 3 Center for Genetic Engineering and Biotechnology, Systems Biology, Havana, Cuba; 4 Center for Genetic Engineering and Biotechnology, Animal housing, Havana, Cuba; 5 Center for Genetic Engineering and Biotechnology, Production Division, Havana, Cuba; Lerner Research Institute - Cleveland Clinic, UNITED STATES

## Abstract

Despite that more than one hundred vaccines against SARS-CoV-2 have been developed and that some of them were evaluated in clinical trials, the latest results revealed that these vaccines still face great challenges. Among the components of the virus, the N-protein constitutes an attractive target for a subunit vaccine because it is the most abundant, highly conserved and immunogenic protein. In the present work, a chimeric protein (N-CD protein) was constructed by the fusion of the N-protein to the extracellular domain of human CD154 as the molecular adjuvant. HEK-293 cells were transduced with lentiviral vector bearing the N-CD gene and polyclonal cell populations were obtained. The N-CD protein was purified from cell culture supernatant and further characterized by several techniques. Immunogenicity studies in mice and non-human primates showed the N-CD protein induced high IgG titers in both models after two doses. Moreover, overall health monitoring of non-human primates demonstrated that animals were healthy during 228 days after first immunization. Data obtained support further investigation in order to develop this chimeric protein as vaccine candidate against COVID-19 and other coronavirus diseases.

## Introduction

Coronavirus disease 2019 (COVID-19), a disease caused by severe acute respiratory syndrome coronavirus-2 (SARS-CoV-2), has become a serious threat to human health causing millions of deaths around the world. SARS-CoV-2 has a ribonucleic acid (RNA) genome, single stranded and positive orientation. This genome is 29.88 Kb and contains approximately 12 open reading frames. Like in all coronaviruses (CoVs), these genes encode four major structural proteins: the nucleocapsid (N) that is associated with the RNA genome and three membrane proteins that are the spike glycoproteins (S), the integral membrane glycoprotein (M) and the envelope protein (E). Protein S contains a receptor-binding domain (RBD) and this RBD domain has an approximate molecular mass of 25 kDa. This domain is involved in viral binding and fusion to the cell membrane, and induces neutralizing antibodies that block binding to the angiotensin II receptor (ACE2) present in host cells. Until now, the RBD domain constitutes the fundamental target for the development of subunit vaccines against SARS-CoV2 [1–3]. The appearance of new circulating variants of the virus due to mutations in the RBD domain has raised important concerns about the geographic and temporal efficacy of vaccines based on this protein domain [4]. In consequence, the design and production of new vaccine antigens against coronavirus remains of interest. Of special attention are those vaccines which prevent infection from earlier post-immunization times with the lowest possible number of doses; and that provide long-term protection by activating the memory response, as well as protection against new strains that are generated due to mutations in the RBD domain. In this context, the N protein constitutes an attractive target for the development of a subunit vaccine because it is highly conserved and immunogenic. In addition, they expressed abundantly throughout the course of the infection [5]. Several studies reported high levels of IgG against SARS-CoV-2 N protein in seropositive patients and suggested this IgM to IgG isotype switch may help to generate more effective antibodies that can inhibit virus infection [6, 7]. Intramuscular vaccination with a SARS‐CoV‐2 N plasmid induced robust antibody production in New Zealand White rabbits and increased the number of interferon‐γ (IFN‐γ) spot‐forming splenocytes in C57BL/6 mice [8]. Intranasal vaccination of BALB/c mice with recombinant adenovirus type‐5 expressing SARS‐CoV‐2 N protein induced CD8 T cell responses in the lung. Also, CD4 T cell responses were observed in the spleen associated with robust antibody production supporting the notion that SARS‐CoV‐2 N protein can work as a target for vaccine development [9].

Subunit vaccines are the most commonly used vaccines because of their safety parameters. Many commercially approved vaccines against diseases caused by viruses such as hepatitis B, influenza, and varicella zoster use this platform [10]. However, because of their lower immunogenicity compared with that of live attenuated vaccines, subunit vaccines need adjuvants, carriers, or engineering of the proteins to boost their immunogenicity. The CD40 molecule is a co-stimulatory molecule found on antigen presenting cells including B cells. This receptor binds its ligand CD154, which is transiently expressed on T cells and other non-immune cells under inflammatory conditions, and activates a set of signals that induce the production of antibodies by B cells. CD154 also plays an important role in the interaction of T cells with other cells of the immune system and in the differentiation of T cells [11]. Various research groups employing multiple strategies [12–15] have explored the use of CD154 as a molecular adjuvant.

Here, we developed a SARS-CoV-2 subunit vaccine candidate (N-CD protein) by fusing the extracellular domain of CD154 (CD40 ligand) with the N protein. The characterization of the chimeric protein, produced in HEK293 cells by lentivirus transduction, confirms the identity and quality of the protein. The evaluation of the immune response induced by its injection in mice and non-human primates (NHPs) revealed that the protein is highly immunogenic which make it an attractive target for vaccine development.

## Materials and methods

### Plasmids

The plasmid pDisplay-CMV-N-CD was constructed by steps. First, the plasmid pDisplay-CMV-linker-CD was generated through the cloning of a linker sequence followed by the gene of the extracellular domain of *Homo sapiens* CD154 (NM_000074.2), as described by Ávalos *et al*. [16] (see also S1 Table for more details). This linker sequence corresponds to a 6 His tail and a linker (Ser-Gly-Gly-Gly-Ser-Gly-Gly-Gly-Gly-Ser-Gly-Gly-Gly-Gly-Ser). Second, a sequence encoding for amino acids (aa) 2–419 of the SARS-CoV-2 N protein was amplified by RT-PCR using mRNA samples extracted from convalescent COVID-19 patients. N protein-F and N protein-R oligonucleotide primers (S1 Table) were designed taking into account the sequence reported for SARS-CoV-2 isolate Wuhan-Hu-1 (GenBank: MN908947.3). The PCR product was cloned into a pGEM-Teasy vector (Promega, Madison, USA) and sequenced. Then, this N gene was amplified by PCR using specific oligonucleotide primers N-Display-F and N-Display-R (S1 Table), adding a *BamHI* restriction site to the 5´ end. Finally, to obtain the plasmid pDisplay-CMV-N-CD, this PCR product was digested with *BamHI* and ligated upstream of the linker sequence in the plasmid pDisplay-CMV-linker-CD digested with the restriction enzymes *BglII* and *SmaI*. In the obtained genetic construction, the N-CD gene is fused at the N-terminus to the murine Ig κ-chain leader sequence, which directs the protein to the secretory pathway.

The other plasmids used in this work correspond to the third generation HIV-1-based lentiviral packaging system (Invitrogen, Carlsbad, USA). To express the N-CD protein through lentiviral system, the lentiviral plasmid pl6twblast [14] was used. The complete expression cassette (hCMV promoter/enhancer + Igk-chain leader sequence + N gene + linker + extracellular domain of CD154 gene) was amplified by PCR from the plasmid pDisplay-CMV-N-CD using the oligonucleotide primers N-pl6-F and hCD154-R (S1 Table), adding a *XmaI* restriction site to the 5´ end. To obtain the plasmid pl6twblast-CMV-N-CD, this PCR product was digested with *XmaI* and ligated in the plasmid pl6twblast digested with the restriction enzymes *XmaI* and *EcoRV*.

### Mammalian cell lines and cell culture conditions

DMEM/F12 medium (Gibco, New York, USA) supplemented with 10% Fetal Bovine Serum (FBS, Capricorn, Ebsdorfergrund, Germany) (DMEM/F12-FBS) was used to culture the original human embryonic kidney cells (HEK-293, ATCC CRL-1573) and FT variant cells (HEK-293FT, ATCC PTA-5077) at 37°C in 5% CO_2_. For the lentivirus production, the HEK-293FT cells were used as packing cells. The lentiviral transduction and recombinant protein expression was performed in HEK-293 cells. The DMEM/F12 culture medium was also used for several procedures, included: cell transduction and production of recombinant protein. Supplementation with FBS or not was performed taking into account the procedural requirements. For the selection of recombinant cells, the drug blasticidin (Gibco, New York, USA) was added to the culture medium at a final concentration of 6 μg/mL.

The evaluation of the effect on cellular growth by the CD154 moiety was carried out in NCI-H460 cell line (ATCC HTB-177). These cells were maintained in DMEM (Gibco, New York, USA), supplemented with 10% FBS, glutamine (300 μg/mL) (Gibco, New York, USA), and penicillin-streptomycin (100 U/mL) (Gibco, New York, USA).

### Plasmid transient expression

Plasmid transient expression was evaluated using a protocol described by Ávalos et al., 2022 [16]. Linear PEI (160 kDa) (Polysciences, Warrington, USA) prepared at 1.0 mg/mL was used at a ratio of (1:1) (PEI: DNA) (w:w). For recombinant protein expression, 10 μg of plasmids bearing the N-CD gene were used and 1 μg of a plasmid bearing green fluorescent protein (GFP) cDNA as positive control of transfection.

### Production and quantification of LVs

For the production of LV bearing the N-CD gene, HEK-293FT cells were transfected using linear PEI (160 kDa) with the plasmid pl6twblast-CMV-N-CD and the helper plasmids from the third generation HIV-1-based LV packaging system (Invitrogen, Carlsbad, USA), as previously described by Lao González *et al*. [17]. The LVs were purified through Lenti-X™ Concentrator kit following manufacturer’s instructions. The LV pellet was diluted in 200–600 μL of DMEM/F12 culture medium and stored at -80°C until use. The titration of the concentrated LV stocks was carried out through an ELISA for the detection of HIV p24 capsid protein (DAVIH-Ag p24, LISIDA, Mayabeque, Cuba).

### Transduction of HEK-293 cells using LV and the generation of N-CD-expressing cell pools

Transduction of HEK-293 cells was performed similar to the method described by Ávalos et al., 2022 [16]. Cells were transduced with values of multiplicities of infection (MOI) of 50 and 100. As a negative control of transduction, cells not incubated with LV but cultured under selection conditions with blasticidin were used. After 6 hours of exposure to LV, 500 μL of selection culture medium 2X (DMEM/F12 supplemented with 20% FBS and 12 μg/mL blasticidin) were added to a final concentration of 10% FBS and 6 μg/mL blasticidin. Another round of transduction was implemented reproducing the procedures described above. After 24 hours, the metabolized culture medium was carefully removed and 1 mL of selection culture medium (DMEM/F12-FBS and 6 μg/mL blasticidin) was added to the negative control and transduced cells. This medium exchange was performed every 48–72 hours for approximately 21 days.

### Evaluation of N-CD expression levels in N-CD-expressing cell pools

To evaluate the N-CD protein-expression levels in cell culture supernatant in static culture, recombinant HEK-293 cell pools (derived from MOI 50 and MOI 100) were seeded in 24-well plates at 0.4 × 10^6^ cells/well in 1 mL of DMEM/F12-FBS culture medium. The experiment was performed in triplicate. Plates were incubated at 37°C in 5% CO_2_ and after 9–10 days cell culture supernatant were harvested to quantify N-CD protein-expression levels by ELISA.

### SDS‑PAGE

Samples of cell culture supernatant or purified N-CD protein, previously precipitated with TCA and sodium deoxicolate or not, were analyzed by SDS-PAGE as described by Laemmli [18]. SARS-CoV-2 N protein (2–419 aa) expressed in *Escherichia coli* (CIGB-Sancti Spiritus, Cuba) was used as positive control and 1–2 μg of protein were applied per gel. Samples and the positive control were loaded in 12.5% SDS-PAGE. After running, gels were used for immunoblotting analysis or stained with Coomassie Brilliant Blue G-250. Precision Plus Protein All Blue Prestained Standard (Bio-Rad, Hercules, USA) or Broad Range Protein Molecular Marker (Promega, Madison, USA) were used as protein standards (10–250 kDa).

### Immunoblotting analysis

Separated proteins by SDS-PAGE were transferred to a nitrocellulose membrane (GE Healthcare, Freiburg, Germany). The membrane was blocked with blocking buffer (5% skim milk in 0.05% Tween 20, pH 7.4) and incubated overnight at 4°C. Afterward, the membrane was rinsed for 5 minutes with washing buffer (0.05% Tween 20, pH 7.4) and this step was repeated 3 times.

For SARS-CoV-2 N-protein detection, the membrane was incubated 1–2 hours at room temperature in shaken conditions with a horseradish peroxidase (HRP)-conjugated mouse anti-SARS-CoV-2 N protein (obtained in *E*. *coli*) monoclonal antibody (1:2000, CBSSNCoV-1-HRP, CIGB-Sancti Spiritus, Cuba) diluted in PBS or with sera from convalescent COVID-19 patients at a dilution 1:250 in blocking buffer, respectively. All individuals gave their informed written consent for the use of their serum for research purposes. The membrane incubated with these sera was washed and incubated for 1 h with HRP-conjugated anti-human IgG 1:10000 (Jackson ImmunoResearch, West Grove, USA, 109-035-098) diluted in blocking buffer as secondary antibody.

The proteins of interest, previously marked by the HRP-conjugated antibodies, were visualized through two different substrates: ECL detection system (GE Healthcare, Buckinghamshire, UK) and/or 3,3’ Diaminobenzidine (DAB).

### Quantification of N-CD protein by ELISA

The quantification of N-CD protein levels was performed by sandwich ELISA. High binding 96-well plates (Costar, New York, USA) were coated with 0.05 μg of anti-SARS-CoV-2 N protein monoclonal antibody (CBSSNCoV-10, CIGB Sancti Spiritus, Cuba) in coating buffer (0.1 M Na_2_CO_3_/NaHCO_3_, pH 9.6). After incubation at 4°C for 16 hours, the plates were washed 3 times with washing buffer (PBS, 0.05% Tween 20, pH 7.4). The samples, diluted in washing buffer, were applied to the plates and incubated at 37°C for 1 hour. After three washes with washing buffer, a HRP-conjugated mouse anti-SARS-CoV-2 N protein (expressed in *E*. *coli*) monoclonal antibody (1:80000, CBSSNCoV-1-HRP, CIGB- Sancti Spiritus, Cuba) was added. After incubation at 37°C for 1 hour, the plates were washed again and a mix of 3,3–5,5-tetramethylbenzidine (TMB) and H_2_O_2_ diluted in citrate-phosphate buffer (pH 5.5) was added as substrate. The reaction was stopped with 2 M H_2_SO_4_ and the optical density (OD) was measured at 450 nm on a microplate reader. For standard curves, SARS-CoV-2 N protein (2–419 aa) (obtained in *E*. *coli*) (CIGB-Sancti Spiritus, Cuba) was used as a standard (0.156 to 5 μg/mL). Samples were analyzed in triplicate.

### Production of N-CD protein in batch culture

The N-CD-expressing HEK-293 cells were seeded in 175 cm^2^ T-flasks at 0.3–0.5 x 10^6^ cells/mL in 25 mL of DMEM/F12-FBS. When the cell monolayer reached 90–100% of cellular confluence, it was carefully rinsed 3 times with PBS and 25 mL of medium DMEM/F12 (without serum) were added for protein harvesting. Cells were incubated at 37°C in 5% CO_2_. Cell culture supernatant was harvested after 10 days and stored at -20°C for further analysis and purification.

### Protein purification

The N-CD protein was purified by metal affinity chromatography (IMAC) using a XK-16/20 column (GE Healthcare, Uppsala, Sweden) packed with Ni-NTA agarose matrix (Qiagen, Valencia, USA) in an AKTA^TM^ pure system (GE Healthcare). The cell culture supernatant containing the N-CD protein was clarified by centrifugation at 4300 g for 10 minutes at 4°C and filtrated through 0.45 μm and 0.2 μm cellulose nitrate filters (Sartorius). Afterward, it was equilibrated to a final concentration of 300 mM NaCl, 50 mM NaH_2_PO_4_ and 10 mM imidazole (equilibrium buffer) and the pH was adjusted to 7.4. The matrix was equilibrated with 5 column volumes (CV) and the supernatant was loaded onto the column overnight at 0.5 mL/min at 4°C. A wash with 10 CV of 300 mM NaCl, 50 mM NaH_2_PO_4_ and 20 mM imidazole, pH 7.4. The elution was done using 5 CV of 300 mM NaCl, 50 mM NaH_2_PO_4_ and 250 mM imidazole, pH 7.4. After purification, a buffer exchange to PBS was done with a PD10 desalting column (GE Healthcare, New York, USA). Protein was concentrated six-fold using Amicon® centrifugal filter units 10K (Millipore, Tullagreen, Ireland). Purified protein was quantified by ELISA as described before.

### In gel-digestion and ESI-MS/MS analysis

#### In-gel tryptic digestion

The N protein sample was applied to 12.5% SDS-PAGE electrophoresis under reducing conditions. In-gel protein staining was carried out with Coomassie Briliant Blue-G250. The major bands observed in the elution step after IMAC purification and recognized by the specific monoclonal antibody in Western Blotting, were sliced and destained by incubation with 250 mM ammonium bicarbonate containing 30% of acetonitrile (v/v). The protein was reduced with 1% 1,4-dithiothreitol and S-alkylated with 2.5% acrylamide. Afterward, each band were washed 3 times with Milli-Q water for 10 min. Then, the slices were cut into small 1 mm^3^ pieces, dried with acetonitrile and rehydrated in a minimum volume of 50 mM ammonium bicarbonate containing trypsin (12.5 ng/L) (Promega, Madison, USA). The digestion was incubated at 37 ˚C for 16 hours. The tryptic digestion was stopped by adding 5% formic acid until pH became acidic. The peptide mixture was desalted by a micro column ZipTip C18 RP (Millipore, Billerica, USA) and eluted in 3.5 μL of 60% acetonitrile and 5% formic acid prior to the ESI-MS analysis.

#### ESI-MS/MS analysis

The tryptic peptides were analyzed with a hybrid orthogonal QToF-2^TM^ tandem mass spectrometer (Micromass, UK). The sample was sprayed using 1200 V for capillary and 35 V for the cone. The ESI-MS was set to the 200–2000 *m/z* range and multiply-charged ions were selected for collision induced dissociation based fragmentation. Collision energies between 20 and 50 eV were used to obtain sequence information in the MS/MS spectrum. Argon was used as a collision gas and the mass spectra were processed by using MassLynx version 4.1 software (Micromass, UK).

### Binding of CD154 to CD40 by ELISA

The binding of CD154 extracellular domain of the N-CD protein to the human CD40 was evaluated by ELISA similar to the procedure described before [16]. High binding 96-well plates (Costar, New York, USA) were coated with 0.25 μg of hCD40-Fc (human secreted CD40, Fc fusion, recombinant protein, Bioscience, San Diego, USA, 71174) in coating buffer (0.1 M Na_2_CO_3_/NaHCO_3_, pH 9.6). Samples of N-CD protein and SARS-CoV-2 N protein produced in *E*. *coli* were serially diluted (1:2 from 100–1.56 μg/mL) in blocking buffer.

### *In vitro* proliferation assay to evaluate CD154 biological activity

The impact of N-CD protein on cell proliferation (NCI-H460 cells) was determined by crystal violet staining [16]. N-CD protein and SARS-CoV-2 N protein expressed in *E*. *coli* (MyBiosource, San Diego, USA, MBS8309646) were added in triplicate at a final concentration of 100 μg/ml. The antitumoral drug CIGB-300 (100 μg/ml) (CIGB, Havana, Cuba) was employed as a positive control of proliferation inhibition of cultured cells. CIGB-300 was dissolved as a 10 mM stock in PBS at room temperature for 5 minutes. For each experiment, a freshly-made stock was used. The drugs were diluted directly into growth medium immediately prior to use.

The cell proliferation percentage was calculated using the formula: cell proliferation (%) = [(OD of treated cells)/ (OD of cell control] x100.

### Binding capability of the N-CD protein to sera from COVID-19 convalescent individuals

Binding capability of the N-CD protein to sera from COVID-19 convalescent individuals was assessed similar to Ávalos et al., 2022 [16]. High binding 96-well plates (Costar, New York, USA) were coated with 0.25 μg of N-CD protein in coating buffer (0.1 M Na_2_CO_3_/NaHCO_3_, pH 9.6). Samples were analyzed in duplicate. As a negative control of this experiment, sera from non-infected individuals were used and diluted in blocking buffer as mentioned above.

All procedures performed in studies involving human participants’ samples were in accordance with the ethical standards of the institutional and/or national research committee and with the 1964 Helsinki declaration and its later amendments or comparable ethical standards. Written informed consent was obtained from all individual participants included in the study.

### Animals and immunization schedules

Animal studies were conducted under the approval of the CIGB Animal Care and Use Committee. Each approved protocol number was cited in the corresponding study as: CICUAL/CIGB/number of the study.

### Immunogenicity in mice

The immunogenicity (CICUAL/CIGB/20099) and a dose study of the N-CD protein (CICUAL/CIGB/2104) were assessed in mice by intramuscular administration of this chimeric antigen. Eight-week-old female BALB/c mice weighing 15–20 grams were divided in experimental groups, each one composed of 10 animals. In both experiments, the placebo group was immunized with PBS. In the immunogenicity study, one group was immunized with 10 μg of the N-CD protein and the other group was the placebo group. In the dose study, there were 4 groups, one corresponding to the placebo and the remaining 3 groups were immunized with 5 μg, 10 μg or 20 μg of the N-CD protein. In both cases, Alhydrogel (Branntag Biosector, Frederikssund, Denmark) was used as the adjuvant at a final concentration of 1.44 mg/mL in 100 μL. The formulation process was carried out at 4°C for 16 hours in orbital shaking. The doses were administered on days 0 and 21. Retro-orbital blood was extracted on days 0 (pre-immune serum), 21 and 35 in the immunogenicity study and, on days 0 (pre-immune serum) and 35 in the dose study, to evaluate specific IgG endpoint titers by ELISA. These samples were incubated at 4°C during 16 hours. Blood clots were separated from serum by centrifugation at 13000 g for 20 min at 4°C. Finally, the serum was collected and stored at -20°C until use. Mice were sedated with 100 mg/Kg of body weight of ketamine hydrochloride to perform the blood extractions.

### Immunogenicity in non-human primates

Three to 4 years old male monkeys (*Macaca fascicularis*) weighing from 2 to 4 kg (CICUAL/CIGB/21004) were divided into 2 experimental groups with 3 animals each one. One group was immunized intramuscularly with 50 μg of the N-CD protein and the placebo group was immunized with PBS. In both cases, Alhydrogel (Branntag Biosector, Frederikssund, Denmark) was used as the adjuvant at a final concentration of 1.44 mg/mL in 500 μL.

Monkeys were exposed to 22–29°C throughout the whole study and housed individually in stainless steel cages (90 cm × 60 cm × 60 cm). Under these conditions, animals saw, heard and smelled other NHP of the same species. They were maintained on a 12/12h light/dark cycle under an environmental enrichment regime with toys and foraging enrichment during 2 h per day. Monkeys were fed with fresh fruits and a commercial diet (granulated formula CMQ 1600 ALYco certified by CENPALAB, Havana, Cuba, containing 25% protein, 3.5% crude fat and 3.8% crude fiber) twice daily at a rate of 150–300 g per monkey according to respective age and body weight. Water was always provided *ad libitum*.

Firstly, monkeys underwent a pre-acceptance process to be included in the study, where a skin test for tuberculin was performed. In addition, monkeys were treated with Ivermectin (200 μg/kg subcutaneously, in the interscapular region of the back). As inclusion criterion, only monkeys without behavioral alteration (stereotyped movements, aggression, self-harm, apathetic/depressed behavior, over-grooming, drinking urine, eating faeces, etc.) and clinically healthy were included in the study.

The formulation process was carried out at 4°C for 16 hours with orbital shaking. The doses were administered on days 0 and 21. Blood draws from the femoral vein were performed after sedation with ketamine hydrochloride (10 mg/Kg) on days -7, 0 (pre-immune serum), 21, 28, 35, 42, 63, 84, 127, 144, 174 and 228 to evaluate specific IgG endpoint titers by ELISA and different hematological and biochemical parameters (CENPALAB, Cuba). Body weight and rectal temperature was measured after sedation throughout the experiment. Once the study was completed, the animals returned to their original colony.

### Evaluation of serum IgG titers by ELISA

Antigen-specific IgG endpoint titers in the sera of immunized animals were measured by ELISA as described by Ávalos *et al*. [16]. In this case, N protein-specific antibodies were detected by coating high binding 96-well plates (Costar, New York, USA) with 0.25 μg of SARS-CoV-2 N protein (expressed in *E*. *coli*) (CIGB-Sancti Spiritus, Cuba). Samples were analyzed in duplicate. Antibody endpoint titer was defined as the reciprocal of the maximum dilution for which the absorbance values corresponding to serum dilutions were greater than twice the absorbance obtained for pre-immune serum.

### Epitope mapping of antibodies against SARS-CoV-2 N protein by ELISA

Epitope mapping was performed by ELISA using 23 linear peptides from SARS-CoV-2 N protein as well as the SARS-CoV-2 N protein obtained in *E*. *coli* (S6 Table). High binding 96-well plates (Costar, New York, USA) were coated with 1 μg of linear peptides in coating buffer (0.1 M Na_2_CO_3_/NaHCO_3_, pH 9.6). The plates were coated with only one peptide in each well in triplicates. After incubation at 37°C for 45 minutes, the plates were washed 3 times with washing buffer (PBS, Tween 0.05%, pH 7.4) and blocked 1 hour at 37°C with blocking buffer (5% skim milk in PBS). The plates were washed again 3 times with washing buffer and sera from immunized monkeys were serially diluted in with washing buffer and added to the plates. Serum from naturally infected or non-infected individuals was used as positive and negative controls, respectively. After an incubation step at 37°C for 1 hour, the plates were washed 5 times with washing buffer and HRP-conjugated anti-human IgG (1:10000, Jackson ImmunoResearch, West Grove, USA, 109-035-098) diluted in washing buffer was added. Then, the plates were incubated at 37°C for 1 hour, washed again and a mix of o-phenylenediamine dihydrochloride (OPD) and H_2_O_2_ diluted in citrate-phosphate buffer (pH 5.0) was added as substrate. The reaction was stopped with 2 M H_2_SO_4_ and absorbance was measured at 492 nm on a microplate reader.

### Microneutralization of live SARS-CoV-2 virus in Vero E6

The neutralization antibody titers were measured by a traditional virus microneutralization assay using SARS-CoV-2 (CUT2010-2025/Cuba/2020 strain). The procedure followed in this case has been previously described by Lazo *et al*. [19]. The highest serum dilution showing an OD value greater than the cut-off was considered as the neutralization titer. The cut-off value is calculated as the average of the OD of the cell control wells divided by 2. The positive control of this assay was pool sera with neutralizing activity obtained from animals immunized with recombinant RBD.

### Statistical analysis

Data was analyzed using Minitab 16.1.1 software (Minitab Inc., United Kingdom). The results are expressed as mean ± standard deviation (SD). The Anderson-Darling test was used to test normality of sample data distribution. Bartlett and Levene tests were used to analyze homoscedasticity. In the case of normality and homoscedasticity were not met, a non-parametric test was implemented. For comparison between N-specific IgG endpoint titers from day 21 and 35 in mice immunized with the N-CD protein, a Mann-Whitney U-test was used because data did not meet the normality and homoscedasticity assumptions. One way-ANOVA was used followed by Tukey test for multiple comparisons among N-specific IgG endpoint titers from NHP serum samples collected at different time points. Results were considered statistically significant at p < 0.05.

## Results

### Functionality of the N-CD expression cassette in the pDisplay-CMV-N-CD and pl6twblast-CMV-N-CD plasmids

Recombinant N-CD protein comprises the SARS-CoV-2 N protein fused by a linker sequence to the extracellular domain of human CD154. Sequencing results showed a different N protein variant compared with Wuhan1 strain. This variant bears an amino acid replacement of RG (AGGGGA) by KR (AAACGA) according a previous report [20].

The Western Blotting analysis of cell culture supernatant of HEK-293 cells transfected with the plasmid pDisplay-CMV-N-CD, using an anti-SARS-CoV-2 N protein monoclonal antibody (S1C Fig) and sera from convalescent COVID-19 patients (S1D Fig), showed a band between 75 and 100 kDa bands of the molecular weight marker, under reducing conditions. The expected molecular weight for N-CD protein is 73,1 kDa. High molecular aggregates were also detected when the electrophoresis was performed in non-reducing conditions. A band around 50 kDa corresponding to SARS-CoV-2 N protein expressed in *E*. *coli*, used as positive control, was also detected.

**Fig 1:**
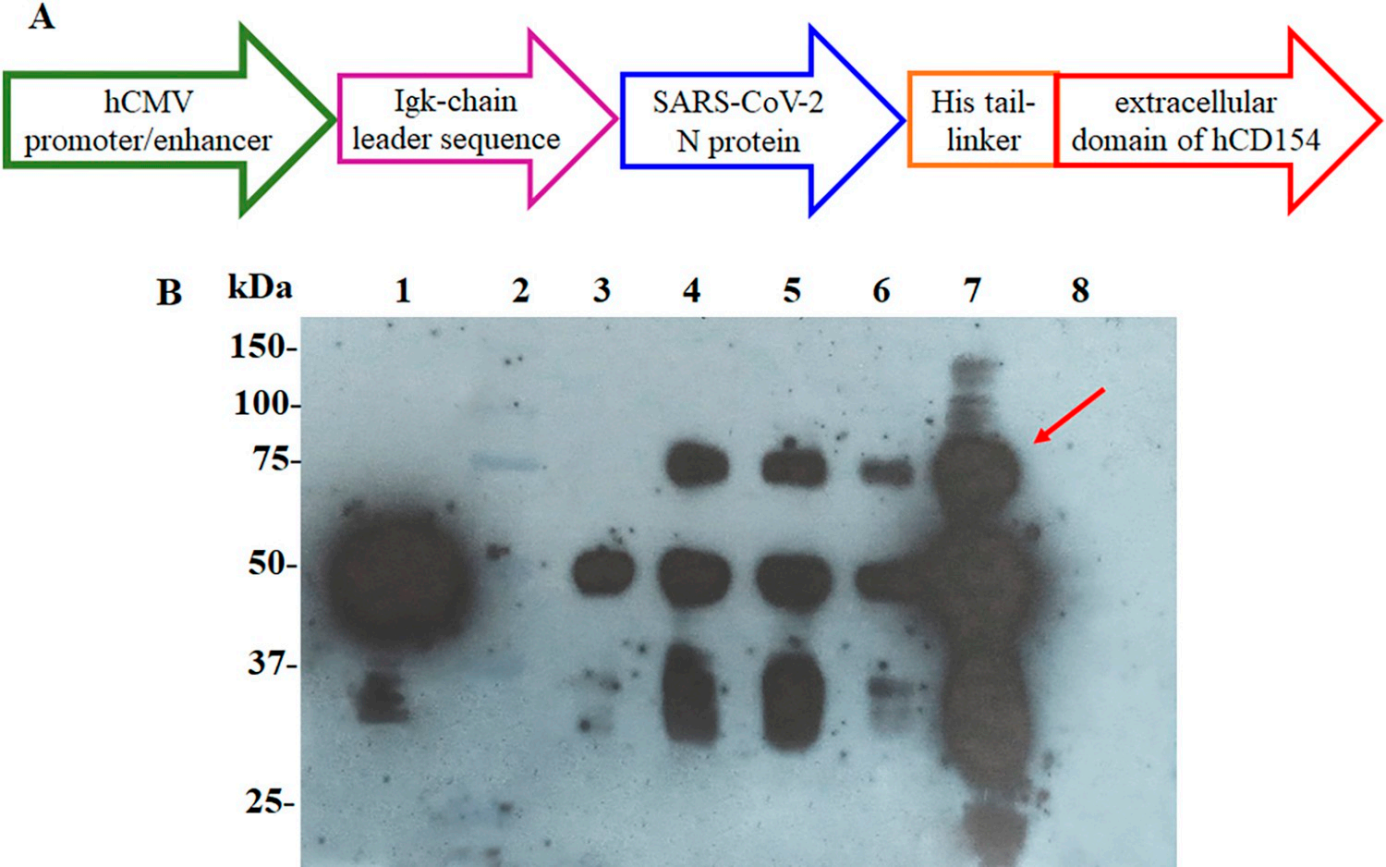
Design and functionality of the N-CD expression cassette cloned into the lentiviral plasmid pl6twblast. **(A)** Schematic diagram of the plasmid pl6twblast-CMV-N-CD (hCMV promoter/enhancer + Igk-chain leader sequence + N gene + 6-His tail + linker + extracellular domain of CD154 gene). **(B)** 750 μL of supernatant of HEK-293 cells transfected with pl6twblast-CMV-N-CD harvested after 72 hours and analyzed under reducing conditions in 12.5% SDS-PAGE. Western Blotting analysis was performed using a HRP-conjugated anti-SARS-CoV-2 N protein monoclonal antibody. Lane 1: SARS-CoV-2 N protein expressed in *E*. *coli*, lane 2: protein standard, lanes 3 to 6: total proteins from supernatant of HEK-293 cells transfected with the lentiviral plasmid pl6twblast-CMV-N-CD (clones 15, 17, 18 and 19, respectively), lane 7: total proteins from supernatant of HEK-293 cells transfected with the plasmid pDisplay-CMV-N-CD (positive control), lane 8: total proteins from supernatant of HEK-293 cells transfected without DNA (negative control). The red arrow points out the non-degraded N-CD protein.

Similar results were obtained in the Western Blotting analysis of the cell culture supernatant of HEK-293 cells transfected with the plasmid pl6twblast-CMV-N-CD (Fig 1B). Under reducing conditions, a band between 75 and 100 kDa was observed in the three clones of pl6twblast-CMV-N-CD analyzed as well as in the supernatant from pDisplay-CMV-N-CD transfected cells (positive control). Moreover, two groups of bands of lower molecular weight were also detected.

### Expression and purification of the N-CD protein

**Fig 2:**
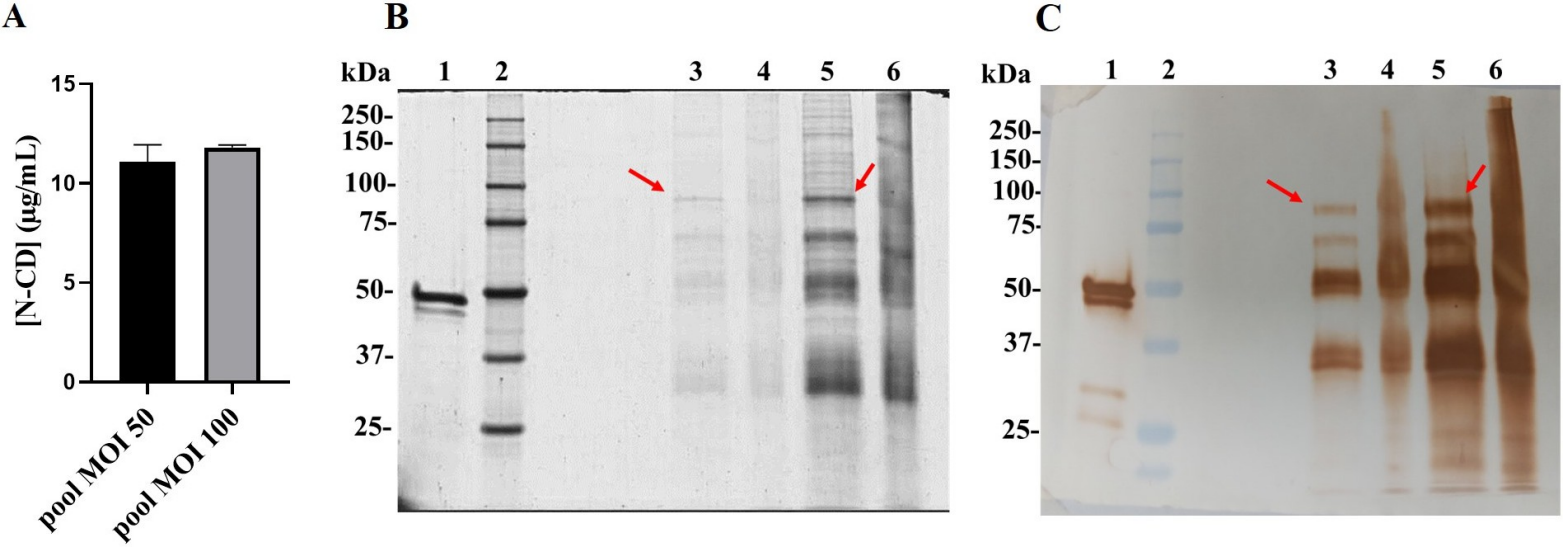
Evaluation of protein expression levels and analysis of purified N-CD protein. (A) N-CD expression in recombinant HEK-293 cell pools (derived from MOI 50 and MOI 100) after 10 days of culture. The data correspond to mean ± standard deviation. (B) SDS-PAGE 12,5% and Coomassie staining and (C) Western blotting using a HRP-conjugated anti-SARS-CoV-2 N protein monoclonal antibody of non-concentrated and six-fold concentrated N-CD protein after IMAC purification analyzed under reducing and non-reducing conditions. This is typical example of several concentration batches. The red arrows point out the non-degraded N-CD protein. Lane 1: SARS-CoV-2 N protein expressed in E. coli, lane 2: protein standard, lane 3: non-concentrated N-CD protein under reducing conditions, lane 4: non-concentrated N-CD protein under non-reducing conditions, lane 5: concentrated six-fold N-CD protein under reducing conditions, lane 4: concentrated six-fold N-CD protein under non-reducing conditions.

Cell pools obtained from HEK-293 cells transduced with MOI 50 (pool-MOI 50) exhibited N-CD protein expression levels by ELISA of approximately 11.09 μg/mL, while cell pool obtained from HEK-293 cells transduced with MOI 100 (pool-MOI 100) presented expression levels of 11.81 μg/mL (Fig 2A). Pool-MOI 50 was selected for the production of the N-CD protein in batch culture due to its faster growth.

The N-CD protein was purified from cell culture supernatant harvested in DMEM/F12 culture medium without serum. Most of the protein as eluted in 250 mM imidazole condition. The main fraction was collected independently and desalted to PBS. Desalted protein was concentrated six-fold and analyzed by SDS-PAGE (Fig 2B, lanes 5 and 6) and Western Blotting (Fig 2C, lanes 5 and 6) showing an increase in protein-band intensity. In this figure (Fig 2C, lanes 3 and 5), we can observe that under reducing conditions the anti-SARS-CoV-2 N protein monoclonal antibody recognizes four groups of bands: one band between 75 and 100 kDa, which probably corresponds to the N-CD protein, and 3 groups of bands with a lower molecular weights (50, 37 and 25–20 kDa, approximately). The same pattern of bands was observed in all the purification batches. For non-reducing conditions, high molecular aggregates were also detected as extra bands with molecular weights higher than 75 kDa (Fig 2C, lanes 4 and 6).

### ESI-MS analysis for the verification of the amino acid sequence

**Fig 3:**
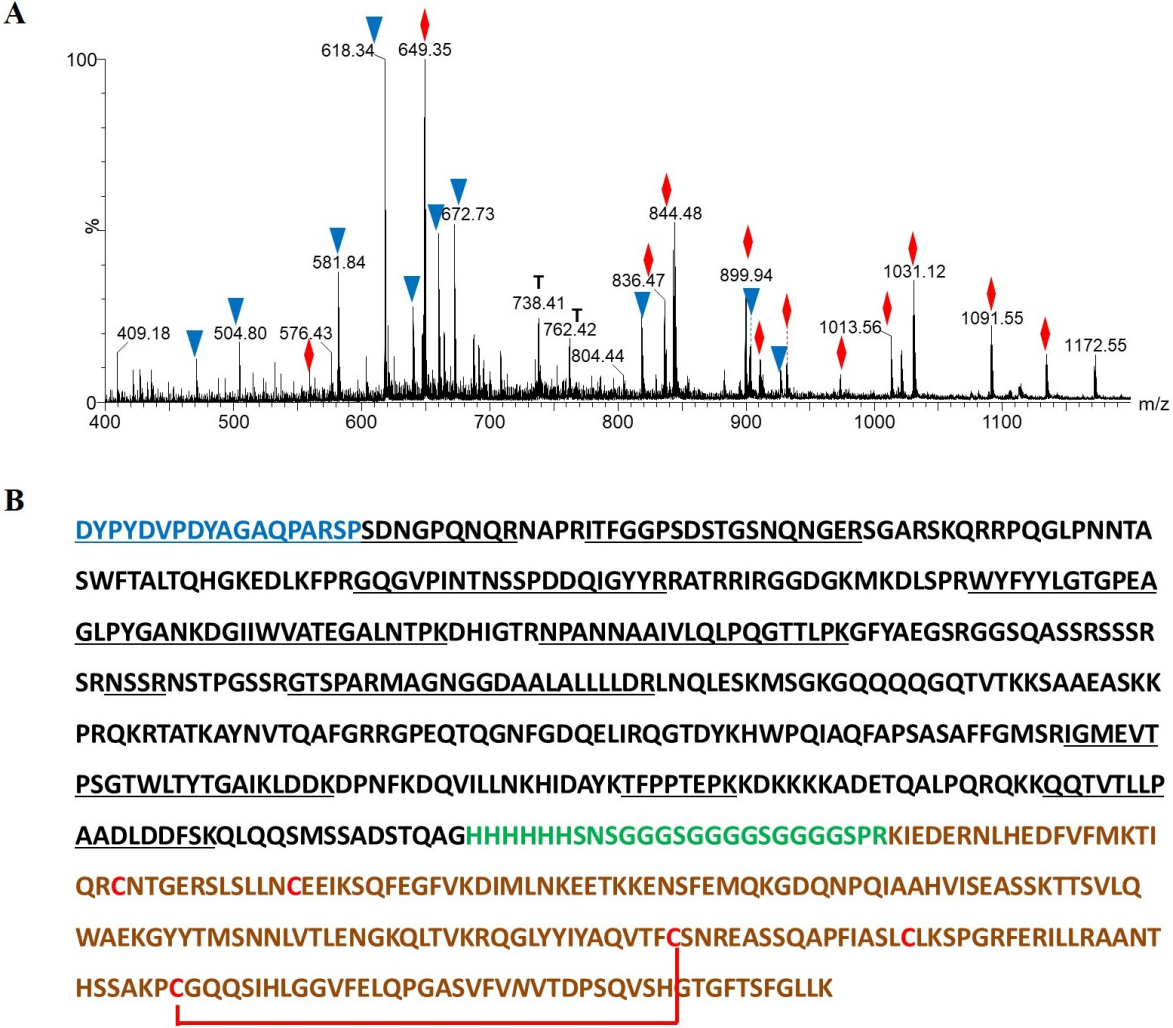
Characterization of the N-CD protein by ESI-MS analysis. The ESI-MS spectrum shown in **(A)** was obtained for the in-gel tryptic digestion of the N-CD protein band that migrated between 75 and 100 kDa according to the molecular weight marker employed. The ion signals labeled with blue triangles correspond to linear peptides derived from the CD154 domain, while the red rhombuses were those detected for the tryptic peptides of the N protein domain **(B)** Amino acid sequence of the N-CD protein. The residues highlighted in blue correspond to the eighteen *N*-terminal amino acids in the expressed N-CD protein introduced by the cloning in pDisplay vector. Spacer arm residues and six tandem histidine residues are written in italics. In black, the domain of the SARS-CoV-2 N protein is indicated. In dark green, the extracellular domain of human CD154 (Lys^52^-Leu^261^). Cysteine residues are highlighted in red. The red lines represent a disulfide bond linking C^178^-C^218^ of CD154. The residues indicated as *N* correspond to the potential *N*-glycosylation sites in the analyzed protein. The underlined amino acids in both domains correspond to the sequence regions verified by ESI-MS analysis.

The N-CD protein expressed in HEK-293 cells has 671 amino acids and 5 Cys residues, all of them present in the extracellular domain of human CD154. Three of Cys residues (Cys^483^, Cys^495^ and Cys^605^) are not linked by disulfide bonds. To confirm the identity of the four bands obtained after purification and concentration (S2A Fig), they were digested *in situ* with trypsin and the proteolytic peptides were extracted and directly analyzed by ESI-MS. The ion signals detected in the ESI-MS showed a good agreement between the expected and the experimental molecular masses for the expected tryptic peptides. The results of ESI-MS/MS analysis are summarized in S2 Table. The sequence coverage obtained for the N-CD protein considering the linear peptides was 50.6% (Fig 3). Peptides from N and the human extracellular domain of CD154 were identified in the four bands analyzed suggesting that lower bands could be degradation products.

### CD154 binding and anti-proliferative effects in H460 lung cells

**Fig 4:**
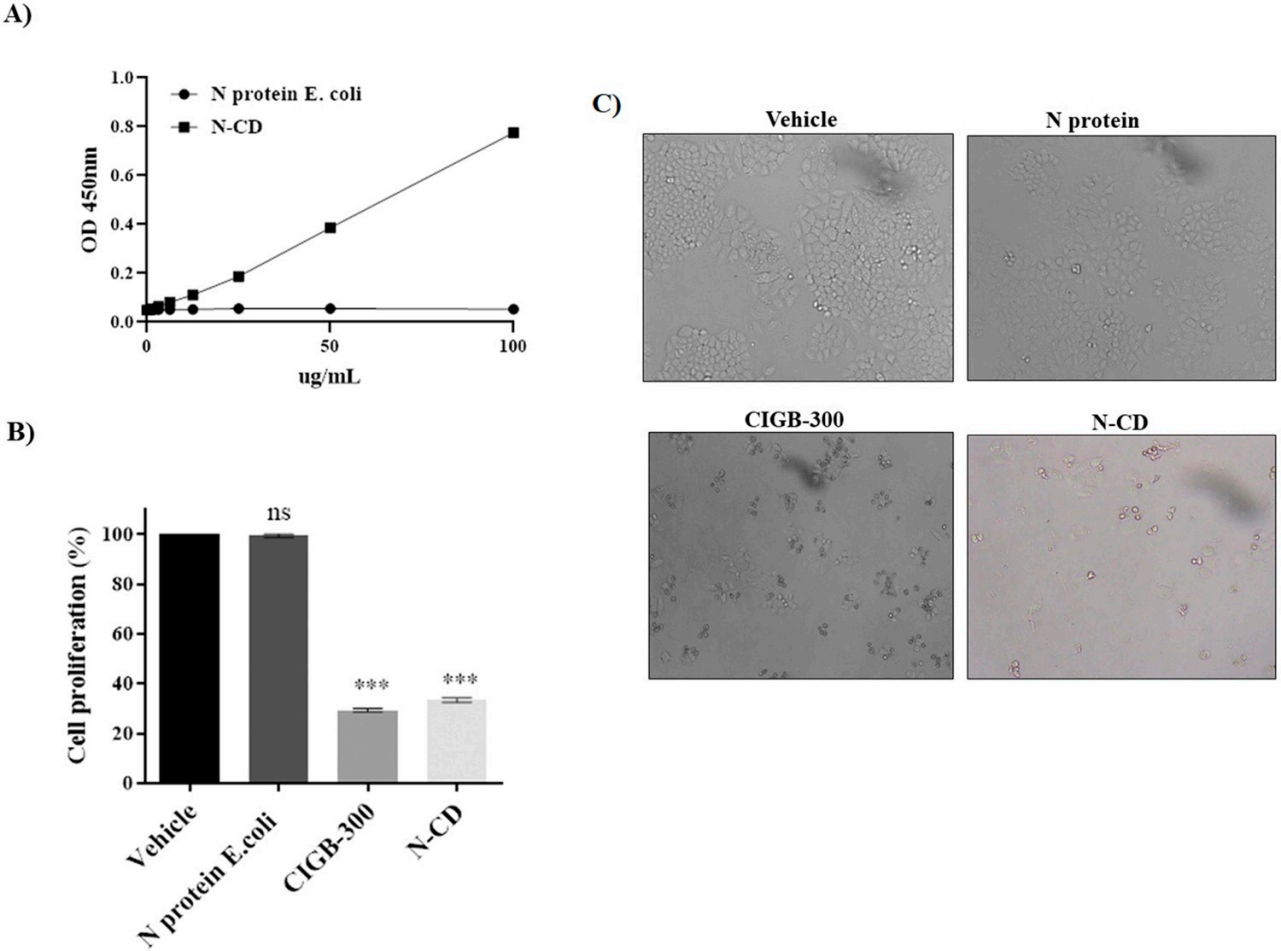
**(A)** Binding of CD154 (CD40L) to CD40 *in vitro*. The binding of CD154 (CD40L) portion of the N-CD protein to the human CD40 was evaluated by ELISA using human CD40-Fc as coating antigen. **(B)** Impact of N-CD on cellular proliferation of the CD40+ lung cancer cell line H460 *in vitro*. The lung cancer cell line was cultured for 72 h with increasing concentrations of N-CD, N protein expressed in *E*. *coli* and CIGB-300 as positive control. Data are shown as mean ± SD, n = 3. Statistically significant differences between vehicle and treatments are represented as *** p < 0.001 and ns, not significant determined using one-way ANOVA followed Dunnett post-test. **(C)** Anti proliferative effect of N-CD in the lung cancer H460 using light microscopy (magnification 10X).

Fig 4A shows that the N-CD protein interacted with the CD40 receptor. On another hand, the absorbance values corresponding to the N protein produced in *E*. *coli* as expected were similar to blanks because this protein lacks CD154 extracellular domain.

The impact of N-CD protein on the growth of large cell lung cancer cell line NCI-H460 was assessed by crystal violet staining. The N-CD protein significantly inhibited the proliferation of the CD40+ cell line NCI-H460 with around 70% of inhibition at 100 μg/ml, while no effect was demonstrated with the N protein expressed in *E*. *coli*. These results confirmed the specific effect of the CD154 moiety present in the chimeric protein (Fig 4B and 4C).

### Characterization of the binding capability of the N-CD protein to sera from convalescent COVID-19 patients

**Fig 5:**
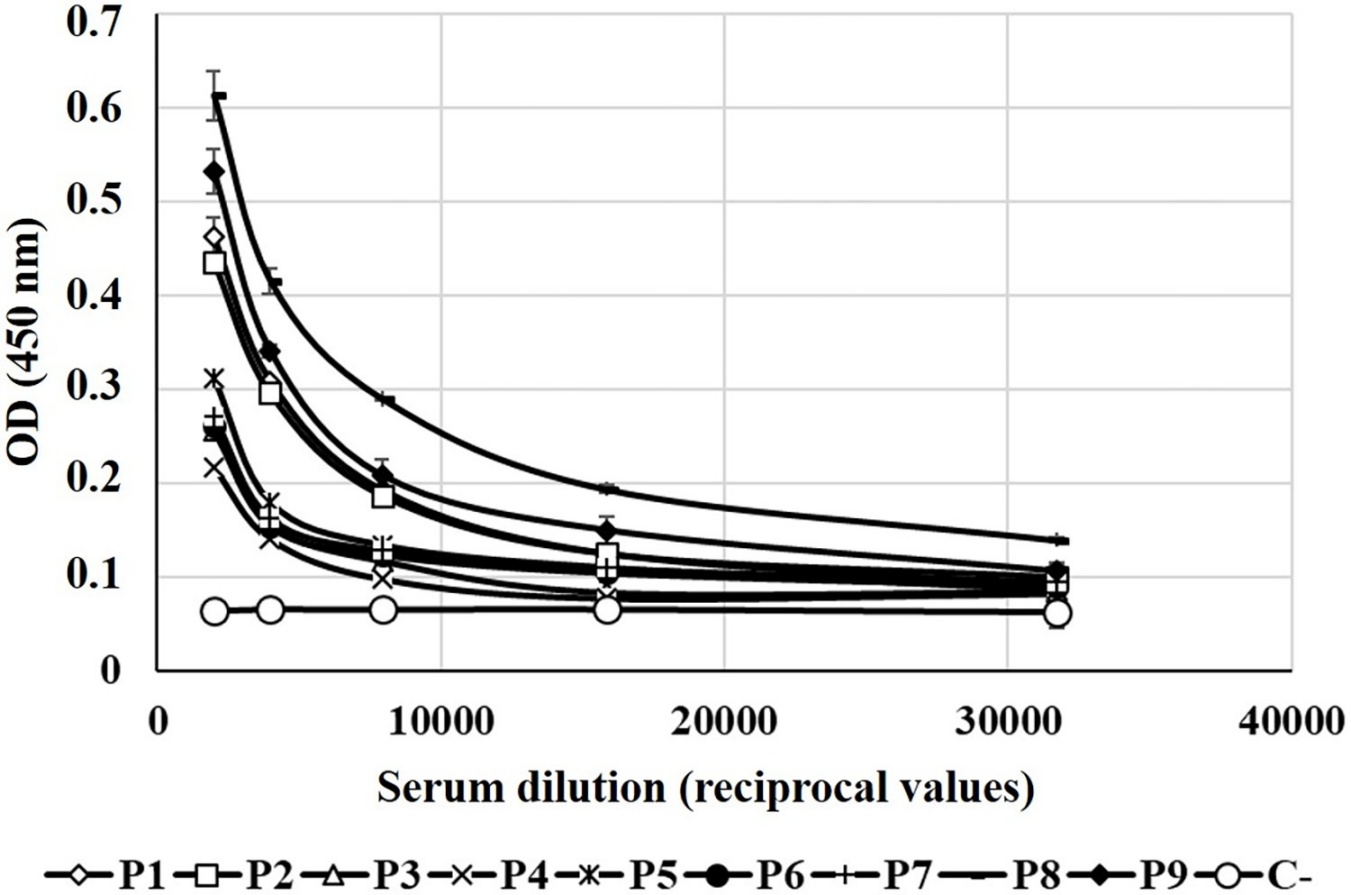
Binding capability of the N-CD protein to sera from convalescent COVID-19 patients. ELISA plates were coated with N-CD protein and incubated with serially diluted sera from convalescent COVID-19 patients. Specific antibodies were detected with HRP-conjugated anti-human IgG. Samples were analyzed in duplicate. Serum from a non-infected individual was used as negative control. The data correspond to mean ± standard deviation.

To evaluate the binding capability of the N-CD protein to sera using samples from previous naturally infected individuals, ELISA plates were coated with the N-CD protein and incubated with sera from patients. Fig 5 shows that the N-CD protein was detected by the antibodies present in the sera from convalescent COVID-19 patients; however, it is not recognized by a serum from a non-infected individual.

### Analysis of humoral immune responses in mice immunized with the N-CD protein

#### Immunogenicity in mice

**Fig 6:**
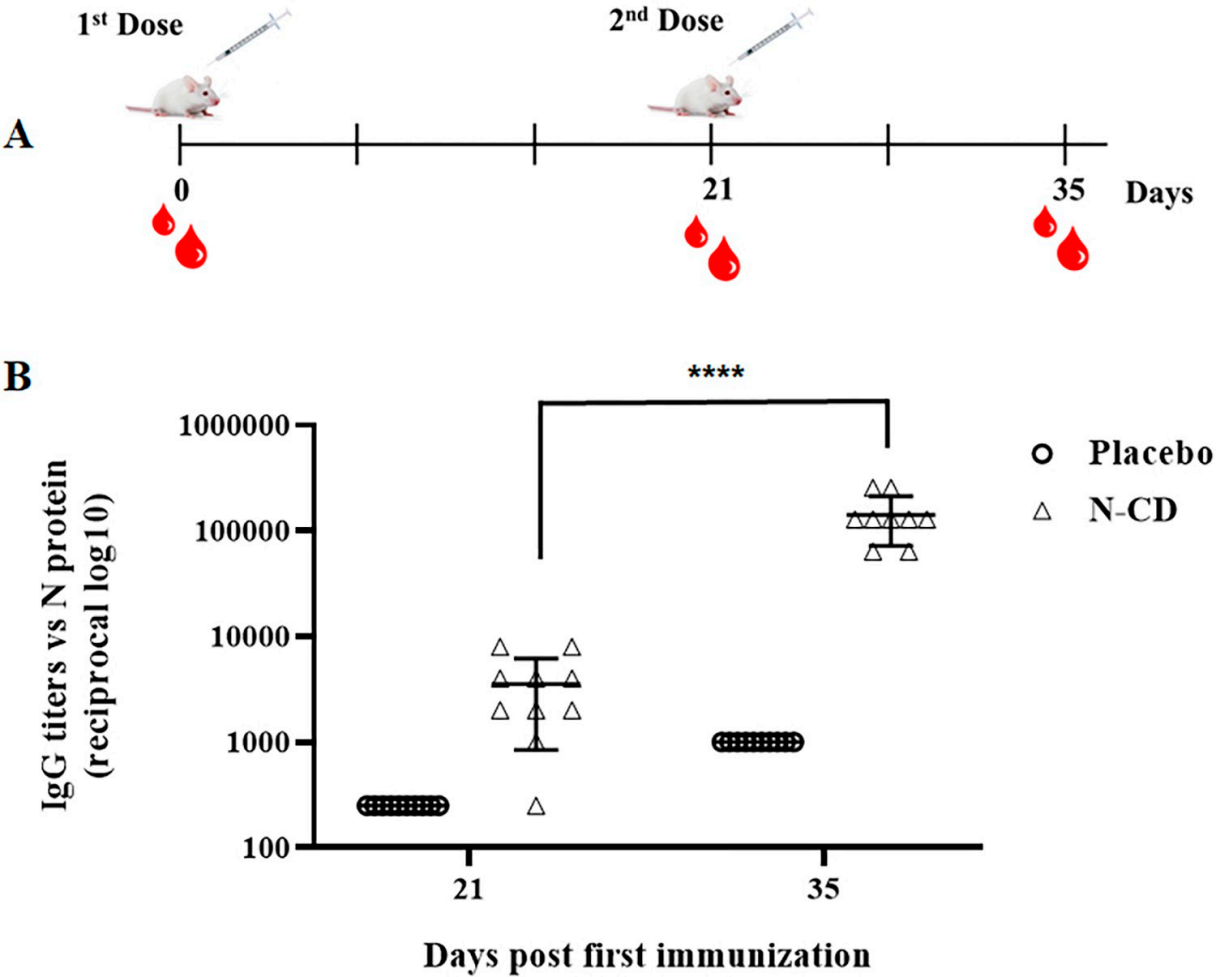
Humoral immune responses induced by N-CD protein in mice. (**A**) Immunization schedule. Mice were intramuscularly immunized with 10 μg of the N-CD protein or PBS (placebo) using alum as an adjuvant on days 0 and 21. Both experimental groups were composed of 10 animals. Blood draws were performed at 0 (pre-immune serum), 21 and 35 after the first immunization. (**B**) N-specific IgG endpoint titers in serum samples collected at 21 and 35 days were measured by ELISA using plates coated with SARS-CoV-2 N protein expressed in *E*. *coli*. The graphic shows mean ± standard deviation. A Mann-Whitney U-test was used for comparison between N-specific IgG endpoint titers from 21 and 35 days. (****) *p* < 0.0001.

In the immunogenicity experiment carried out in BALB/c mice (Fig 6), the immunization of animals with the first dose of the N-CD protein was able to induce 100% of seroconversion and mean IgG endpoint titers of 3525 ± 2683. The administration of a booster at day 21 significantly impacted IgG endpoint titers; indeed, 35 days post first immunization the mean of IgG endpoint titers was 142222 ± 69946.

The results of the dose response experiment in mice showed that IgG endpoint titers increased with increasing dose and the best values compared to placebo were obtained with the dose of 20 μg per animal (131200 ± 91074) compared with 5 μg (60800 ± 38311) and 10 μg (139200 ± 147811), but no statistical differences were found among N-CD experimental groups (S3 Fig).

### Analysis of humoral immune responses in monkeys immunized with N-CD protein

#### Humoral immune response in monkeys

**Fig 7:**
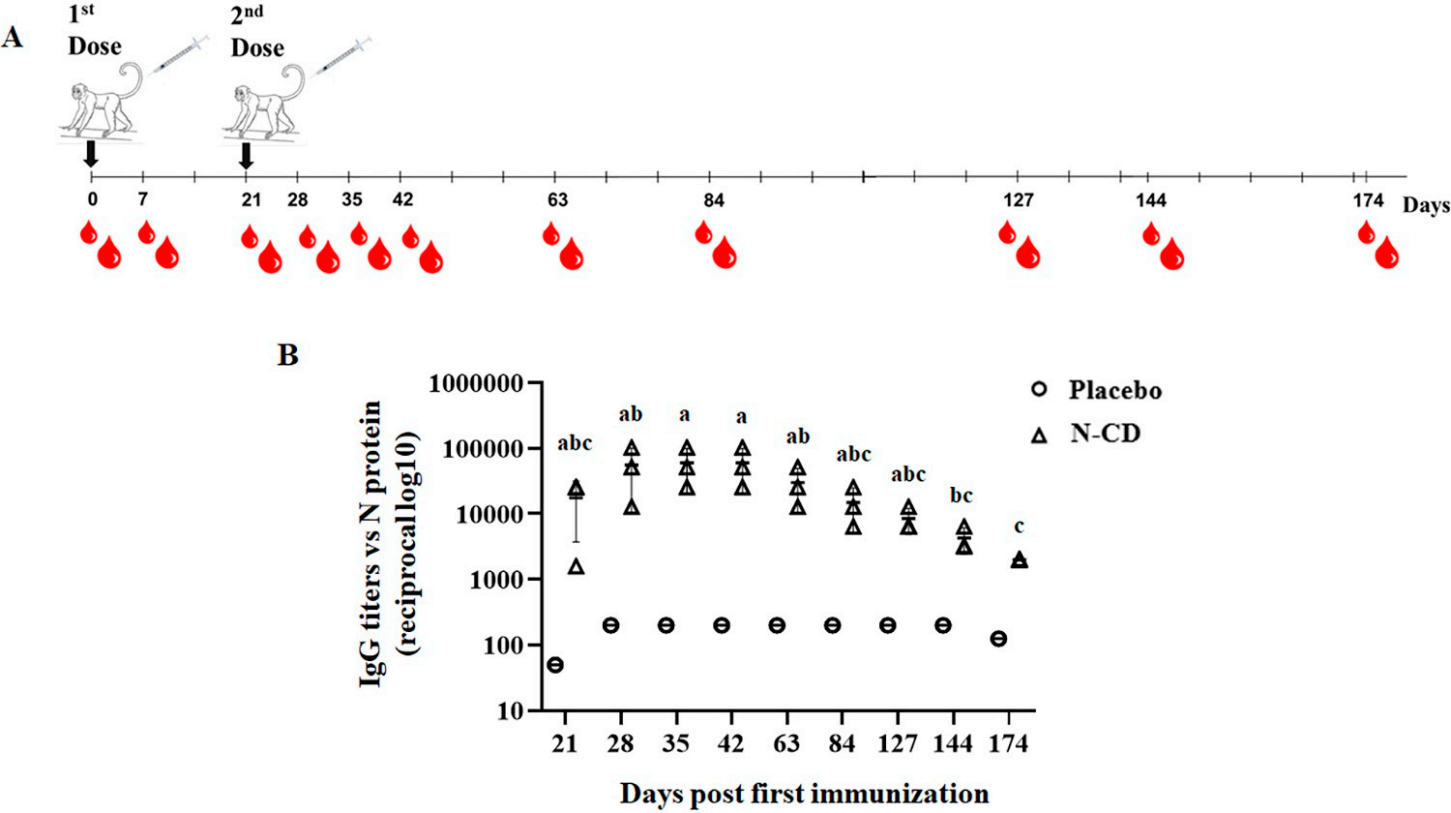
Humoral immune responses induced by N-CD protein in monkeys. (A) Overall immunization schedule. Monkeys were intramuscularly immunized with 50 μg of the N-CD protein or PBS (placebo) using alum as an adjuvant on days 0 and 21. Both experimental groups were composed of 3 animals. Blood draws were performed at 0 (pre-immune serum) and at 9 different times during almost 6 months. (B) N-specific IgG endpoint titers at 21, 28, 35, 42, 63, 84, 127, 144 and 174 days were measured by ELISA using plates coated with SARS-CoV-2 N protein expressed in *E*. *coli*. The graphic shows mean ± standard deviation. One way-ANOVA followed by Tukey test for multiple comparisons were used for comparison between N-specific IgG endpoint titers from serum samples collected at different times after the immunization with the N-CD protein or placebo. Different letters indicate significant differences (p < 0.01) between different timepoints.

The immunization of monkeys with N-CD protein induced an antibody response at 21 days after the first dose, with a mean of IgG endpoint titers of 17600 ± 13856 and seroconversion of 100%. The administration of a booster at day 21 increased IgG endpoint titers. Indeed, 42 days after the first immunization (21 days after the second dose) the mean of IgG endpoint titers were 59733 ± 39104 and started to decline at day 63 with IgG endpoint titers of 29867 ± 19552. After 174 days (5.8 months approximately) of the first immunization, the 3 animals presented mean IgG endpoint titers of 2000 (Fig 7).

#### Epitope mapping of antibodies against SARS-CoV-2 N protein by ELISA

Sera from monkeys immunized with the N-CD protein collected at day 35 post first immunization were used for antibody epitope mapping. Results showed that the antibodies present in the sera from the three immunized monkeys reacted with a peptide located in the C-tail of the SARS-CoV-2 N protein (Table 1). The results related to the other 22 peptides are depicted in S6 Table.

**Table 1:** Epitope mapping of antibodies against SARS-CoV-2 N protein by ELISA. The absorbance values obtained from the assessment of the sera from monkeys immunized with the N-CD protein or the placebo group, using a peptide from SARS-CoV-2-N protein or the complete protein as coating antigen, are showed. A positive result was considered when the mean absorbance values were higher than 0.18 (media plus three times the standard deviation of absorbance values of negative control). Positive control: Serum from convalescent COVID-19 patient. Negative control: Serum from non-infected patient. Blank control: wells were filled only with washing buffer.

Coating antigen	Domain	C-tail peptide	Complete SARS-CoV-2 N protein
	Sequence	PKKDKKKKADETQALPQRQKK	
**Experimental groups**	Placebo-1	0.08	0.07
	Placebo-2	0.08	0.09
	Placebo-3	0.10	0.07
	N-CD-1	0.48	0.83
	N-CD-2	0.21	0.78
	N-CD-3	0.23	0.80
**Assay controls**	Control +	0.41	0.88
	Control -	0.08	0.08
	Blank	0.05	0.06

### Microneutralization of live SARS-CoV-2 virus in Vero E6

A pool of sera from day 35 was used to evaluate the neutralization titer of live SARS-CoV-2 virus in Vero E6 cells. The findings indicate that the serum from immunized animals with the N-CD protein do not to interfere with the direct entrance of the virus into the cells because neutralization titers were not detected with the lowest serum dilution used in the assay (1:20). However, pool sera from animals immunized with RBD, the positive control of this assay, showed a neutralization titer of 80.

### Overall health monitoring of monkeys

Monkeys were monitored throughout the experiment to check their health. Body temperature in the animals was in the range of 37 and 39.5°C (S4B Fig) which is the normal temperature range in cynomolgus macaques [21]. Moreover, body weight was stable throughout the experiment (S4C Fig). The evaluation of hematological and biochemical parameters from blood samples collected before start the experiment, at 42 days of the beginning of the experiment and at the end (228 days) of the experiment (S4A Fig), showed that animals were healthy (S3–S5 Tables).

## Discussion

Since the emergence of SARS-CoV-2 variants with enhanced transmissibility, such as alpha, beta, delta and omicron (https://www.who.int/en/activities/tracking-SARS-CoV-2-variants/); there is a growing concern that these new variants could impair the efficacy of current vaccines [22]. Indeed, the variants present mutations that are found in the antigenic supersite in the N-terminal domain of the spike protein or in the ACE2-binding site of the RBD, which is a major target of potent virus-neutralizing antibodies elicit by vaccines [22–24]. Taking into account this evidence, many scientists have proposed to use the N protein of SARS-CoV-2 as an important target for vaccine development [8, 9, 25–27]. New vaccines candidates based on the combination of Spike protein or RBD and N protein, could control SARS-CoV-2 in a more efficient manner. The combination of both antigens may elicit T cell and neutralizing antibody responses that cross-react with different SARS-CoV-2 variants [28–32].

Commercial available SARS-CoV-2 N protein is mainly produced in *E*. *coli*, but it has also been produced in insect cells or HEK-293 cells [33]. In this work, we propose the production of a chimeric vaccine antigen in HEK-293 mammalian cells. The decision is supported by the distinctive advantage of mammalian cells generating close to wild-type environments for transcription and translation; coupled with the relevant chaperone, secretory, and redox environments and post-translational modifications that favor proper protein folding and lead to functionally relevant and active proteins [34].

Glycosylation in the chimeric protein can explain an increased apparent mass [35, 36] observed in Western Blotting under reducing conditions using a monoclonal antibody against SARS-CoV-2 N protein for detection. The band corresponding to this protein was between 75 and 100 kDa of the molecular weight marker used, although the predicted molecular weight of the N-CD protein is 73.1 KDa. A recent glycoproteomic study of SARS-CoV-2 N protein transiently expressed in HEK-293 cells confirmed the presence of *N*-glycosylation on two of five consensus sequons. These were modified with either high-mannose or complex type *N*-glycans, and *O*-linked glycosylation in addition to phosphorylation at an unexpected location (Thr393), which occurred in the secretory pathway [33]. Additionally, human CD154 contains a single N-linked glycosylation site at asparagine 240 [37].

We observed that purified N-CD protein was degraded in four well-defined groups of bands (75, 50, 37 and 25–20 kDa. Similar results were obtained by Supekar et al. [33]. These authors did not provide any explanation about why these 4 bands were observed. We hypothesized that degradation was a result of the activity of proteases in the culture medium and the autoproteolytic property of the N protein. Supernatant was harvested 10 days after the serum-free culture medium was added to cells. Despite cell monolayer was washed several times with PBS to eliminate serum before to adding serum-free culture medium, serum contaminants could remain and present proteases that cleaved the N-CD protein [38]. Preliminary results of cell adaptation to a protein-free culture medium and suspension culture obtained by us showed a significant decrease in the degradation of the N-CD protein.

A recent study showed a detail study of the autoproteolytic activity of recombinant SARS-CoV-2 N protein [39]. Three distinct protein bands were detected at ~49, ~38 and ~28 kDa for the N protein in SDS-PAGE. Then, the mass of five proteoforms were identified that ranged from 22.6 to 42.9 kDa [39]. This finding could also explain our results, taking into account they work with a protein expressed in *E*. *coli*, while we probably obtained a glycosylated N protein portion, which cause an apparent molecular mass shifts by glycosylation. The specificity of cleavage at conserved residues identified in that study without a common motif, suggest that there may be a structural component directing autoproteolysis. The authors also identified various stoichiometries of N proteoforms that are influenced by pH. They suggested that cleavage of N protein is likely a feature of coronavirus infection and is not unique to SARS-CoV-2 N protein [39].

The results indicated that N-CD protein is capable of binding to its cellular receptor CD40. These results suggest that the N protein fused at the N-terminal of CD154 does not affect its interaction with the receptor and the CD154 extracellular domain is correctly folded. A previous study reported that CD40 signaling induced growth inhibition of CD40-positive lung cancer cells [40]. Based on this, the CD154 within the chimera can triggers the signaling mechanism after CD154-CD40 interaction, resulting in the inhibition of NCI-H460 cell proliferation as it was observed. CIGB-300, an anticancer peptide, was used as a positive control of the experiment since it is reported that it inhibits tumor cell proliferation *in vitro* [41, 42]. No CD40 reactivity in ELISA or cell proliferation inhibition was observed with N protein produced in *E*. *coli* as expected due to the absent of the CD154 fragment.

The N-CD protein was able to form high molecular weight aggregates, as showed in western blotting under non-reducing conditions, probably due to the ability of N protein to self-associate and form oligomers (dimer, trimer, tetramer, or hexamers), that is a crucial property for genome encapsidation [25, 43, 44]. Furthermore, the extracellular structure of CD154 favors the characteristic trimerization of TNF superfamily members [45]. The oligomerization forming two or more trimers (multi-trimers) is critical for the optimal activity of CD154 [46], which may enhance the aggregation of the N-CD protein. This property of the N-CD protein could promote its immunogenicity. Regardless of the safety of protein subunit vaccines, their low immunogenicity raises the necessity of booster doses and the addition of an adjuvant to achieve stronger and more durable immunization [47]. N-CD protein was highly immunogenic in two different animal models. In BALB/c mice, the intramuscularly administration of 10 μg of N-CD protein at day 0 and 21, induced IgG endpoint titers against the SARS-CoV-2 N protein principally of 10^3^ to 10^4^ at day 21 after the first immunization. Two weeks after the second dose, anti‐N IgG endpoint titers reached values of 10^5^. Twenty-one days after the first administration of 50 μg of N-CD protein through intramuscular route in NHP, anti‐N IgG endpoint titers were around 10^3^ and 10^4^. The second dose of N-CD protein at day 21, elicited an antibody response with anti‐N IgG endpoint titers of 10^4^ to 10^5^ and those values were maintained for almost 9 weeks after the second dose. In a similar experiment, Mamedov *et al*. [48] immunized BALB/c mice intramuscularly on days 0 and 21 with 5 μg of a recombinant N protein transiently produced in *Nicotiana benthamiana* plants and adjuvated in alum. In this case, IgG endpoint titers specific to the N protein had values around 10^6^ and 10^7^ in sera collected on days 21 and 42 after injection, respectively [48]. Those IgG titers observed in our experiments were very similar to those obtained with more immunogenic approaches such as DNA vaccines or adenovirus vectors. Ahlén *et al*. [8] immunized intramuscularly six New Zealand White rabbits with 0.3 or 0.9 mg of a SARS-CoV-2 N plasmid at weeks 0 and 3 using *in vivo* electroporation (EP). A single injection of the N plasmid induced anti-N titers of 10^3^ to 10^4^ at week 2, increasing up to 10^4^ to 10^5^ at 2 weeks after a boost (week 5), with no difference in the DNA dose used [8]. In another example, BALB/c mice were primed and boosted (6–7 weeks after priming) by intranasal route with 1 × 10^7^ plaque‐forming‐units of recombinant adenovirus type‐5 expressing SARS‐CoV‐2 N protein (Ad5‐N) or Ad5‐empty. Five days post boosting anti‐N IgG titers were around 10^4^ to 10^5^ in the serum of immunized mice [9].

The sera from NHP immunized with the N-CD protein did not exhibit virus neutralizing activity. The same results were observed by Mamedov *et al*. [48] with sera from mice immunized with N protein obtained in *N*. *benthamiana* plants [48]. Therefore, the humoral response against this antigen could protect individuals from SARS-CoV-2 infection through other mechanisms. Epitope mapping showed that N-specific antibodies (IgG) from NHP immunized with the N-CD protein mainly recognized the C-tail fragment adjacent to the N protein CTD domain. It has been reported that the middle or C-terminal region of the SARS-CoV N protein is important for eliciting antibodies against SARS-CoV during the immune response [49–51]. Recently, a healthcare worker cohort study demonstrated that the presence of anti-N SARS-CoV-2 antibodies (acquired after natural infection) at baseline not only reduced the risk of positive nasopharyngeal SARS-CoV-2 tests, but also the occurrence of COVID-19 symptoms among participants during almost 8 months [52]. Díez et al. observed that SARS-CoV-2 hyperimmune immunoglobulin from human convalescent plasma induced ADCC (Antibody-Dependent Cell-Mediated Cytotoxicity) and ADCP (Antibody-dependent cellular phagocytosis) against SARSCoV-2 N and S [53], suggesting that role of antibodies against SARS-CoV-2 N protein in these mechanisms could be a determining factor in resolving SARS-CoV-2 infections through an S-protein independent mechanism involving host immune system cells [53]. Anti-nucleoprotein antibodies can facilitate particle and antigen uptake and presentation, leading to reduced viral titers and morbidity [54, 55]. Finally, Dangi et al. provided the first demonstration in the coronavirus literature that antibody responses specific to the nucleocapsid protein can improve viral clearance. They first immunized mice with a nucleocapsid-based vaccine and then transferred sera from these mice into naive mice, followed by challenge with SARS-CoV-2 [56]. They showed that mice that received nucleocapsid-specific sera or a nucleocapsid-specific mAb exhibited enhanced control of SARS-CoV-2. Although nucleocapsid-specific antibodies failed to neutralize cell-free SARS-CoV-2 *in vitro*, they and others have recently shown that nucleocapsid protein is expressed on the surface of SARS-CoV-2–infected cells [57, 58], and their findings suggested that nucleocapsid-specific antibodies conferred antiviral protection via recognition of infected cells displaying the nucleocapsid antigen on their surface, which triggered NK-mediated ADCC against infected cells [56]. Beyond neutralization and nucleocapsid-specific T cell responses, the nucleocapsid-specific humoral responses during SARS-CoV-2 infection may play a role in the protection from and/or resolution of SARS-CoV-2 infection through other IgG Fc-dependent functionalities, especially relevant for the treatment of more neutralization-resistant SARS-CoV-2 variants.

Evidence from the literature indicates that the targeted delivery of an antigenic protein (either HIV or SARS-CoV-2 proteins) to CD40 positive cells enhance the immune response, allowing for an early protection against infection. This was accomplished by fusing the antigenic protein to the antibody specific for CD40 [59]. In this work, we propose a protein-based vaccine, in which the N protein of SARS-CoV-2 is fused to the CD40 ligand (CD154) in place of the antibody against CD40. This should assure the delivery of the antigen directly to the antigen presenting cells and should reduce the need for specific adjuvants stimulating the innate response allowing for early protection upon immunization. A similar chimeric design to the one used in this work was developed before in our research department to produce a chimeric RBD-CD antigen [16]. RBD-CD protein was also tested in BALB/c mice and NHP animal models. Human CD154 extracellular domain shares 74.64% and 99.52% identity with the murine and macaque CD154 extracellular domain, respectively. The RBD-CD results in these animal models support their use in the animal experiments developed to evaluate N-CD. Additionally, an enhancement of humoral and cellular host immune response of RBD-CD compared to RBD alone produced in HEK-293 cells was demonstrated, and NHP sera neutralized the live virus infection to Vero E6 cells only after two doses of RBD-CD protein. This validated the use of CD154 as molecular adjuvant. The NHP weight, temperature and biochemical blood test monitoring during almost 7 months after the first immunization, demonstrated that animals were healthy during all the experimental time for both proteins N-CD and RBD-CD [16].

Altogether, the results obtained support the further generation of individual clones of N-CD transformed cells and their adaptation to growth in cell suspension culture with the objective to avoid or reduce protein degradation and to obtain an N-CD protein with higher integrity. Once this goal is achieved, a comparison experiment with SARS-CoV-2 N protein produced under the same conditions will be developed to study the adjuvant effect of CD154 which is a limitation of current study. Future research goals will be the development of additional analysis for antibody function, the evaluation of T cell response and immunization/challenge experiments to prove vaccination effectiveness *in vivo*.

The N-CD protein produced in HEK-293 cells could be used in combination with RBD-based vaccines; for example, with the Cuban Abdala vaccine, which is based on a RDB protein expressed in *Pichia pastori*s and adjuvated in alum [60], to modulate immune response and enhances protection against different SARS-CoV-2 variants but further experimentation will be necessary to confirm the efficacy of this combinatory approach.

## Supporting information

S1 Fig Design and functionality of the N-CD expression cassette. **(A)** Schematic diagram of the N-CD expression cassette (hCMV promoter/enhancer + Igk-chain leader sequence + N gene + 6-His tail + linker + extracellular domain of CD154 gene). **(B)** Full sequence of the N-CD expression cassette. Simple underlined letters: Ig κ-chain leader sequence. Bold letters: SARS-CoV-2 N protein. Simple underlined and bold letters: amino acid replacement of RG (AGGGGA) by KR (AAACGA). Cursives letters: 6 His tail. Double underlined letters: Gly-Ser linker. Bold and cursive letters: extracellular domain of human CD154. Simple underlined and cursive letters: stop codon. Non-highlighted sequence corresponds to residues from pDisplay backbone. Supernatant of HEK-293 cells transfected with the plasmid pDisplay-CMV-N-CD was harvested after 72 hours. Total proteins from 750 μL of supernatant were precipitated and analyzed under reducing and non-reducing conditions in 12.5% SDS-PAGE. For Western Blotting analysis, proteins were transferred to a nitrocellulose membrane and immunodetection of N protein was performed using a HRP-conjugated anti-SARS-CoV-2 N protein monoclonal antibody (**C**) and sera from convalescent COVID-19 patients (**D**). ECL detection system was used as substrate. **C**: lane 1: SARS-CoV-2 N protein expressed in *E*. *coli*, lane 2: protein standard, lane 3: total proteins under reducing conditions, lane 4: total proteins under non-reducing conditions. **D**: lane 1: protein standard, lane 2: total proteins under non-reducing conditions, lane 3: total proteins under reducing conditions, lane 4: SARS-CoV-2 N protein expressed in *E*. *coli*.
(DOCX)

S2 Fig ESI–MS analysis for the in gel digestion protocols and sequence coverage of N-CD protein. ESI–MS analysis for the in gel digestion protocols and sequence coverage of N-CD protein. **(A)** SDS-PAGE of samples from purification process analyzed under reducing conditions in 12,5% gel. Sliced bands used for ESI–MS analysis are indicated in black charts. Lane a: SARS-CoV-2 N protein expressed in E. coli, lane b: protein standard, lane c: equilibrated supernatant, lane d: pass, lane e: equilibrium/washing step at 20 mM imidazole condition, lane f: washing step at 20 mM imidazole condition, lane g: peak of the washing step at 20 mM imidazole condition, lane h: peak of the elution at 250 mM imidazole condition (fraction 1), lane i: peak of the elution at 250 mM imidazole condition (fraction 2). ESI–MS analysis for the in gel digestion protocols and sequence coverage of N-CD protein. **(B)** ESI-MS spectrum for the tryptic in gel digestion of the four gel bands indicated in (**A)**. The signals marked with blue charts were those linear peptides detected for the CD154 domain and the red charts those detected for the N protein domain.
(DOCX)

S3 Fig Dose-response study in mice. Dose-response study in mice. (**A**) Overall immunization schedule. Mice were intramuscularly immunized with 5, 10 or 20 μg of the N-CD protein or PBS (placebo) using alum as an adjuvant on days 0 and 21. The experimental groups were composed by 10 animals each one. Blood draws were performed at 0 (pre-immune serum) and 35 after the first immunization. (**B**) N-specific IgG endpoint titers at 35 days were measured by ELISA using plates coated with SARS-CoV-2 N protein expressed in *E*. *coli*. Serum samples were serially diluted from 1:1000 to 1:2048000. The titer was defined as the highest dilution which presents an optical density that is twice the value of the corresponding pre-immune serum. The graphic shows mean ± standard deviation. Kruskal-Wallis test followed by Dunn’s multiple comparisons test were used for comparisons between IgG endpoint titers from different experimental groups. (*) p < 0.05. (***) p < 0.001. (****) p < 0.0001.
(DOCX)

S4 Fig Overall health monitoring of monkeys. Overall health monitoring of monkeys. **(A)** Animals were intramuscularly immunized with 50 μg of the N-CD protein or PBS (placebo) using alum as an adjuvant on days 0 and 21. Both experimental groups were composed by 3 animals. Blood draws were performed at -7, 42 and 228 days for hematological and biochemical tests. **(B)** and **(C)** Body temperature and weight, respectively, measured at different days during the experimentation schedule in monkeys.
(DOCX)

S5 Fig Multiple sequence alignment of extracellular domain of CD154 from different species. **(A)** Protein sequences of mouse, macaque, swine and human extracellular domain of CD154 were aligned using the bioinformatic tool Clustal Omega 2.1 (https:// www.ebi.ac.uk/Tools/msa/clustalo/). **(B)** Percent identity matrix of the aligned sequences. (*) Positions with a single, fully conserved residue. (:) Positions with conservation between amino acid groups of similar properties. (.) Positions with conservation between amino acid groups of weakly similar properties.
(DOCX)

S6 Fig Original images for SDS-PAGE and Western Blot. (DOCX)

S1 Table List of oligonucleotides used for N gene isolation and cloning. (DOCX)

S2 Table Summary of the ESI–MS analysis for the in gel digestion protocols and sequence coverage of N-CD protein showed in S2 Fig. (DOCX)

S3 Table Blood biochemical data for monkeys seven days before the first immunization. Nd (not determined).
(DOCX)

S4 Table Blood biochemical data for monkeys 42 days after the first immunization. Nd (not determined).
(DOCX)

S5 Table Blood biochemical data for monkeys 228 days after the first immunization. (DOCX)

S6 Table Epitope mapping of antibodies against SARS-CoV-2 N protein by ELISA. The absorbance values obtained from the assessment of the sera from monkeys immunized with the N-CD protein or the placebo group, using a peptide from SARS-CoV-2-N protein or the complete protein as coating antigen is showed. The absorbance values of the assay controls such as positive, negative and blank controls are also depicted in this table. A positive result was considered when the mean absorbance values were higher than 0.18 (media plus three times the standard deviation of absorbance values of negative control). Positive control: Serum from convalescent COVID-19 patient. Negative control: Serum from non-infected patient. Blank control: wells were filled only with washing buffer.
(DOCX)

S1 Data Excel sheets with the raw data of binding of CD154 to CD40 by ELISA, binding of N-CD to sera from COVID-19 convalescent individuals, ELISA results of immunogenicity in mice at days 21 and 35 post first immunization, dose experiments in mice with 5, 10 and 20 μg of N-CD protein for immunization and ELISA results of immunogenicity in monkeys (days 21, 28, 35, 42, 63, 84, 127, 144, 174 post first immunization). (XLSX)
