# Supplementary material for: Production and characterization of a chimeric antigen, based on nucleocapsid of SARS-CoV-2 fused to the extracellular domain of human CD154 in HEK-293 cells as a vaccine candidate against COVID-19
Source: PLoS One. 2023 Sep 26;18(9):e0288006. doi: 10.1371/journal.pone.0288006 (PMC10522030; doi:10.1371/journal.pone.0288006)
Supplement: S1 Fig — (A) Schematic diagram of the N-CD expression cassette (hCMV promoter/enhancer + Igk-chain leader sequence + N gene + 6-His tail + linker + extracellular domain of CD154 gene). (B) Full sequence of the N-CD expression cassette. Simple underlined letters: Ig κ-chain leader sequence. Bold letters: SARS-CoV-2 N protein. Simple underlined and bold letters: amino acid replacement of RG (AGGGGA) by KR (AAACGA). Cursives letters: 6 His tail. Double underlined letters: Gly-Ser linker. Bold and cursive letters: extracellular domain of human CD154. Simple underlined and cursive letters: stop codon. Non-highlighted sequence corresponds to residues from pDisplay backbone. Supernatant of HEK-293 cells transfected with the plasmid pDisplay-CMV-N-CD was harvested after 72 hours. Total proteins from 750 μL of supernatant were precipitated and analyzed under reducing and non-reducing conditions in 12.5% SDS-PAGE. For Western Blotting analysis, proteins were transferred to a nitrocellulose membrane and immunodetection of N protein was performed using a HRP-conjugated anti-SARS-CoV-2 N protein monoclonal antibody (C) and sera from convalescent COVID-19 patients (D). ECL detection system was used as substrate. C: lane 1: SARS-CoV-2 N protein expressed in E. coli, lane 2: protein standard, lane 3: total proteins under reducing conditions, lane 4: total proteins under non-reducing conditions. D: lane 1: protein standard, lane 2: total proteins under non-reducing conditions, lane 3: total proteins under reducing conditions, lane 4: SARS-CoV-2 N protein expressed in E. coli. (DOCX) [file pone.0288006.s001.docx]

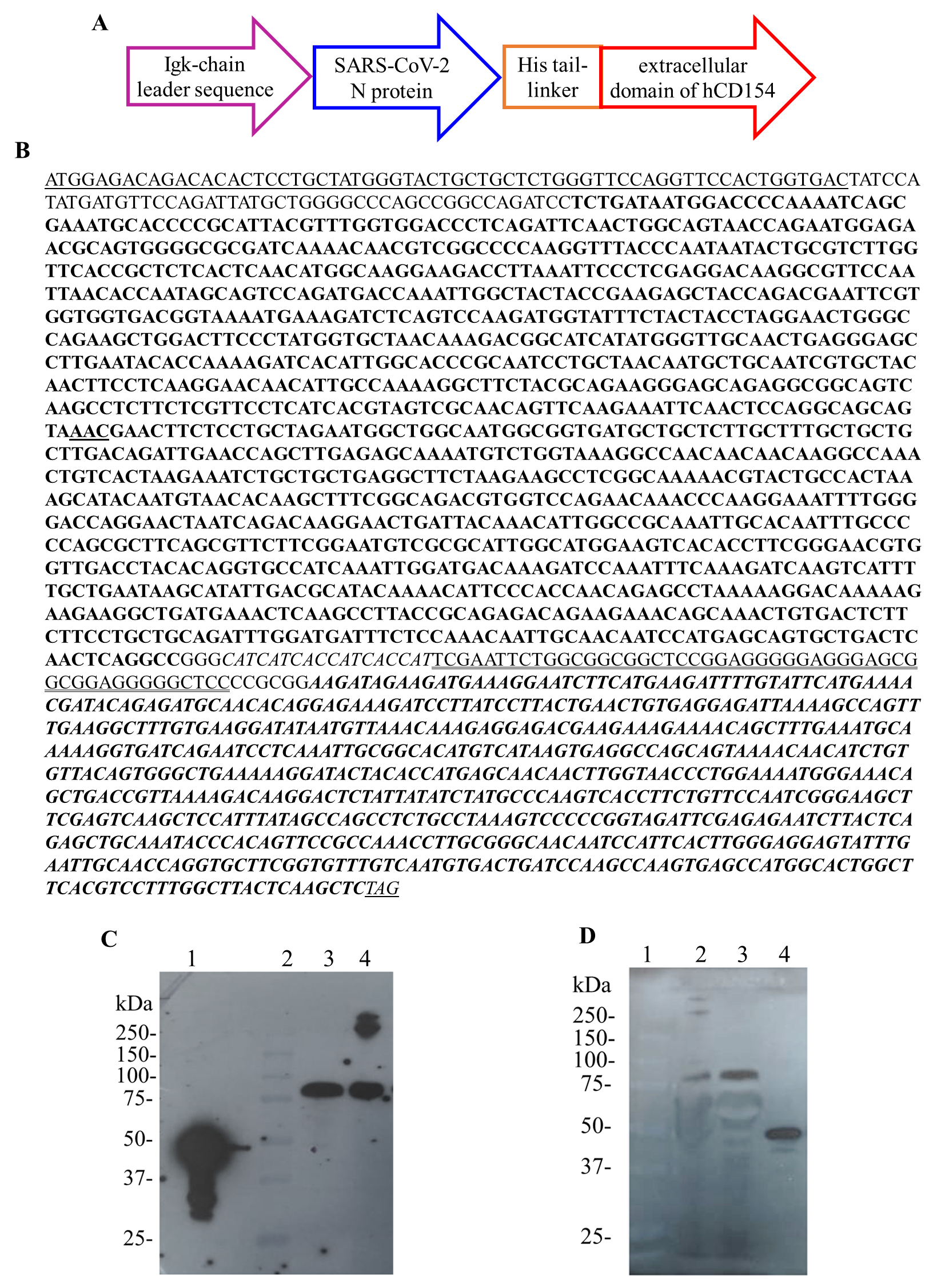


**Supplemental Fig. 1** Design and functionality of the N-CD expression cassette. **(A)** Schematic diagram of the N-CD expression cassette (hCMV promoter/enhancer + Igk-chain leader sequence + N gene + 6-His tail + linker + extracellular domain of CD154 gene).  **(B)** Full sequence of the N-CD expression cassette. Simple underlined letters: Ig κ-chain leader sequence. Bold letters: SARS-CoV-2 N protein. Simple underlined and bold letters: amino acid replacement of RG (AGGGGA) by KR (AAACGA). Cursives letters: 6 His tail. Double underlined letters: Gly-Ser linker. Bold and cursive letters: extracellular domain of human CD154. Simple underlined and cursive letters: stop codon. Non-highlighted sequence corresponds to residues from pDisplay backbone. Supernatant of HEK-293 cells transfected with the plasmid pDisplay-CMV-N-CD was harvested after 72 hours. Total proteins from 750 µL of supernatant were precipitated and analyzed under reducing and non-reducing conditions in 12.5 % SDS-PAGE. For Western Blotting analysis, proteins were transferred to a nitrocellulose membrane and immunodetection of N protein was performed using a HRP-conjugated anti-SARS-CoV-2 N protein monoclonal antibody (**C**) and sera from convalescent COVID-19 patients (**D**). ECL detection system was used as substrate. **C**: lane 1: SARS-CoV-2 N protein expressed in *E. coli*, lane 2: protein standard, lane 3: total proteins under reducing conditions, lane 4: total proteins under non-reducing conditions. **D**: lane 1: protein standard, lane 2: total proteins under non-reducing conditions, lane 3: total proteins under reducing conditions, lane 4: SARS-CoV-2 N protein expressed in *E. coli.*
