# Supplementary material for: Production and characterization of a chimeric antigen, based on nucleocapsid of SARS-CoV-2 fused to the extracellular domain of human CD154 in HEK-293 cells as a vaccine candidate against COVID-19
Source: PLoS One. 2023 Sep 26;18(9):e0288006. doi: 10.1371/journal.pone.0288006 (PMC10522030; doi:10.1371/journal.pone.0288006)
Supplement: S2 Fig — ESI–MS analysis for the in gel digestion protocols and sequence coverage of N-CD protein. (A) SDS-PAGE of samples from purification process analyzed under reducing conditions in 12,5% gel. Sliced bands used for ESI–MS analysis are indicated in black charts. Lane a: SARS-CoV-2 N protein expressed in E. coli, lane b: protein standard, lane c: equilibrated supernatant, lane d: pass, lane e: equilibrium/washing step at 20 mM imidazole condition, lane f: washing step at 20 mM imidazole condition, lane g: peak of the washing step at 20 mM imidazole condition, lane h: peak of the elution at 250 mM imidazole condition (fraction 1), lane i: peak of the elution at 250 mM imidazole condition (fraction 2). ESI–MS analysis for the in gel digestion protocols and sequence coverage of N-CD protein. (B) ESI-MS spectrum for the tryptic in gel digestion of the four gel bands indicated in (A). The signals marked with blue charts were those linear peptides detected for the CD154 domain and the red charts those detected for the N protein domain. (DOCX) [file pone.0288006.s002.docx]

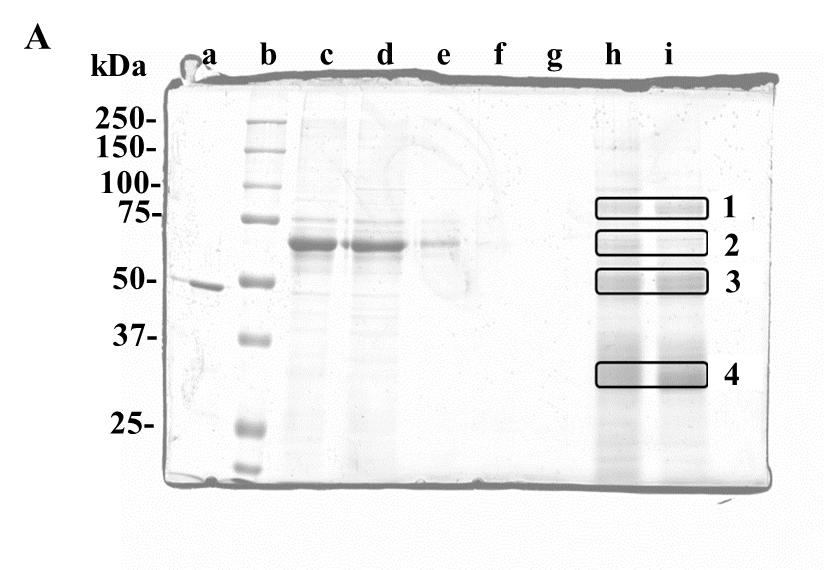


**Supplemental Fig. 2** ESI–MS analysis for the in gel digestion protocols and sequence coverage of N-CD protein. **(A)** SDS-PAGE of samples from purification process analyzed under reducing conditions in 12,5 % gel. Sliced bands used for ESI–MS analysis are indicated in black charts. Lane a: SARS-CoV-2 N protein expressed in E. coli, lane b: protein standard, lane c: equilibrated supernatant, lane d: pass, lane e: equilibrium/washing step at 20 mM imidazole condition, lane f: washing step at 20 mM imidazole condition, lane g: peak of the washing step at 20 mM imidazole condition, lane h: peak of the elution at 250 mM imidazole condition (fraction 1), lane i: peak of the elution at 250 mM imidazole condition (fraction 2)


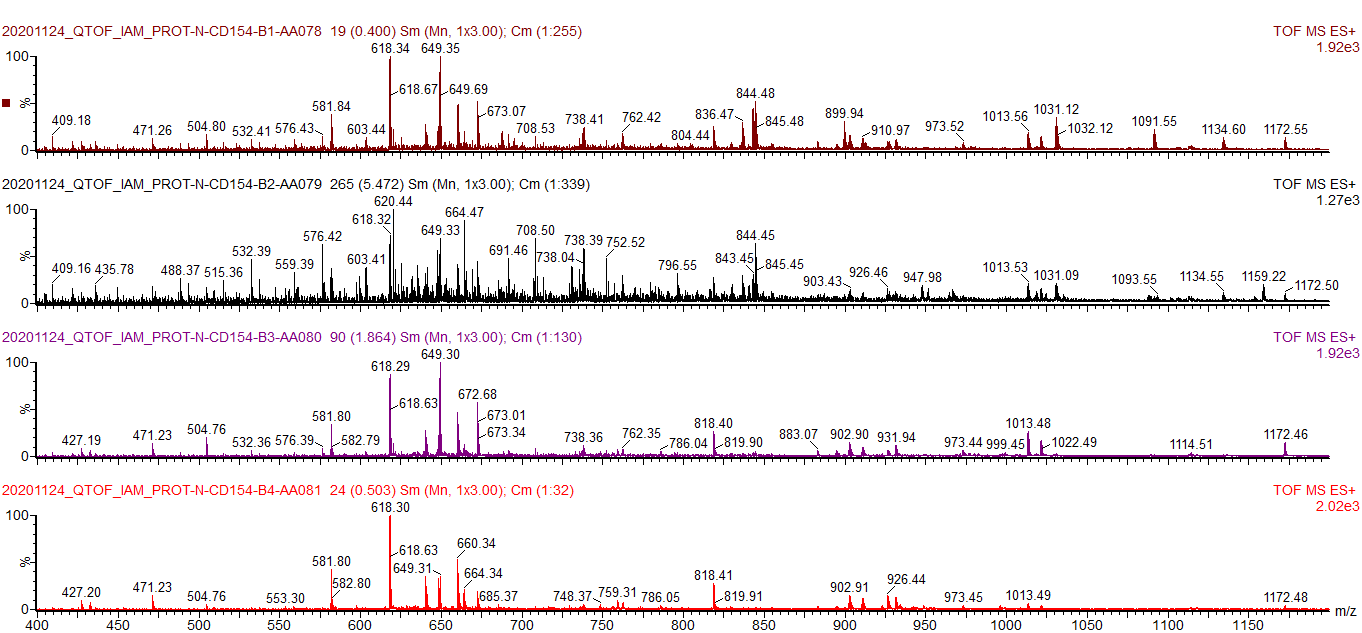


**Band 3**

**B**

**Band 4**

**Band 2**

**Band 1**

**Supplemental Fig. 2 (continuation)** ESI–MS analysis for the in gel digestion protocols and sequence coverage of N-CD protein. **(B)** ESI-MS spectrum for the tryptic in gel digestion of the four gel bands indicated in (**A)**. The signals marked with blue charts were those linear peptides detected for the CD154 domain and the red charts those detected for the N protein domain
