# Supplementary material for: Production and characterization of a chimeric antigen, based on nucleocapsid of SARS-CoV-2 fused to the extracellular domain of human CD154 in HEK-293 cells as a vaccine candidate against COVID-19
Source: PLoS One. 2023 Sep 26;18(9):e0288006. doi: 10.1371/journal.pone.0288006 (PMC10522030; doi:10.1371/journal.pone.0288006)
Supplement: S3 Fig — Dose-response study in mice. (A) Overall immunization schedule. Mice were intramuscularly immunized with 5, 10 or 20 μg of the N-CD protein or PBS (placebo) using alum as an adjuvant on days 0 and 21. The experimental groups were composed by 10 animals each one. Blood draws were performed at 0 (pre-immune serum) and 35 after the first immunization. (B) N-specific IgG endpoint titers at 35 days were measured by ELISA using plates coated with SARS-CoV-2 N protein expressed in E. coli. Serum samples were serially diluted from 1:1000 to 1:2048000. The titer was defined as the highest dilution which presents an optical density that is twice the value of the corresponding pre-immune serum. The graphic shows mean ± standard deviation. Kruskal-Wallis test followed by Dunn’s multiple comparisons test were used for comparisons between IgG endpoint titers from different experimental groups. (*) p < 0.05. (***) p < 0.001. (****) p < 0.0001. (DOCX) [file pone.0288006.s003.docx]

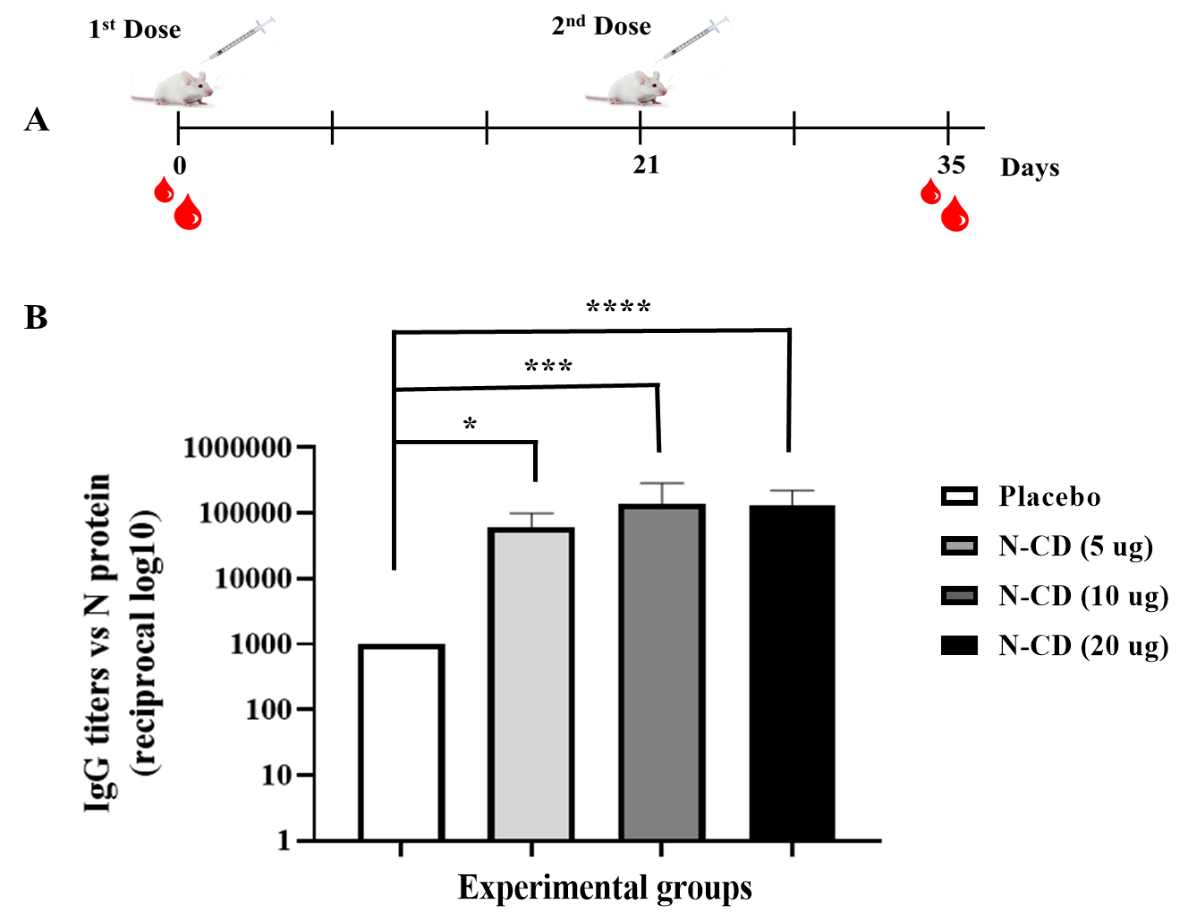
**Supplemental Fig. 3** Dose-response study in mice. (**A**) Overall immunization schedule. Mice were intramuscularly immunized with 5, 10 or 20 µg of the N-CD protein or PBS (placebo) using alum as an adjuvant on days 0 and 21. The experimental groups were composed by 10 animals each one. Blood draws were performed at 0 (pre-immune serum) and 35 after the first immunization. (**B**) N-specific IgG endpoint titers at 35 days were measured by ELISA using plates coated with SARS-CoV-2 N protein expressed in *E. coli*. Serum samples were serially diluted from 1:1000 to 1:2048000. The titer was defined as the highest dilution which presents an optical density that is twice the value of the corresponding pre-immune serum. The graphic shows mean ± standard deviation. Kruskal-Wallis test followed by Dunn's multiple comparisons test were used for comparisons between IgG endpoint titers from different experimental groups. (*) p < 0.05. (***) p < 0.001. (****) p < 0.0001.
