# Supplementary material for: Production and characterization of a chimeric antigen, based on nucleocapsid of SARS-CoV-2 fused to the extracellular domain of human CD154 in HEK-293 cells as a vaccine candidate against COVID-19
Source: PLoS One. 2023 Sep 26;18(9):e0288006. doi: 10.1371/journal.pone.0288006 (PMC10522030; doi:10.1371/journal.pone.0288006)
Supplement: S4 Fig — Overall health monitoring of monkeys. (A) Animals were intramuscularly immunized with 50 μg of the N-CD protein or PBS (placebo) using alum as an adjuvant on days 0 and 21. Both experimental groups were composed by 3 animals. Blood draws were performed at -7, 42 and 228 days for hematological and biochemical tests. (B) and (C) Body temperature and weight, respectively, measured at different days during the experimentation schedule in monkeys. (DOCX) [file pone.0288006.s004.docx]

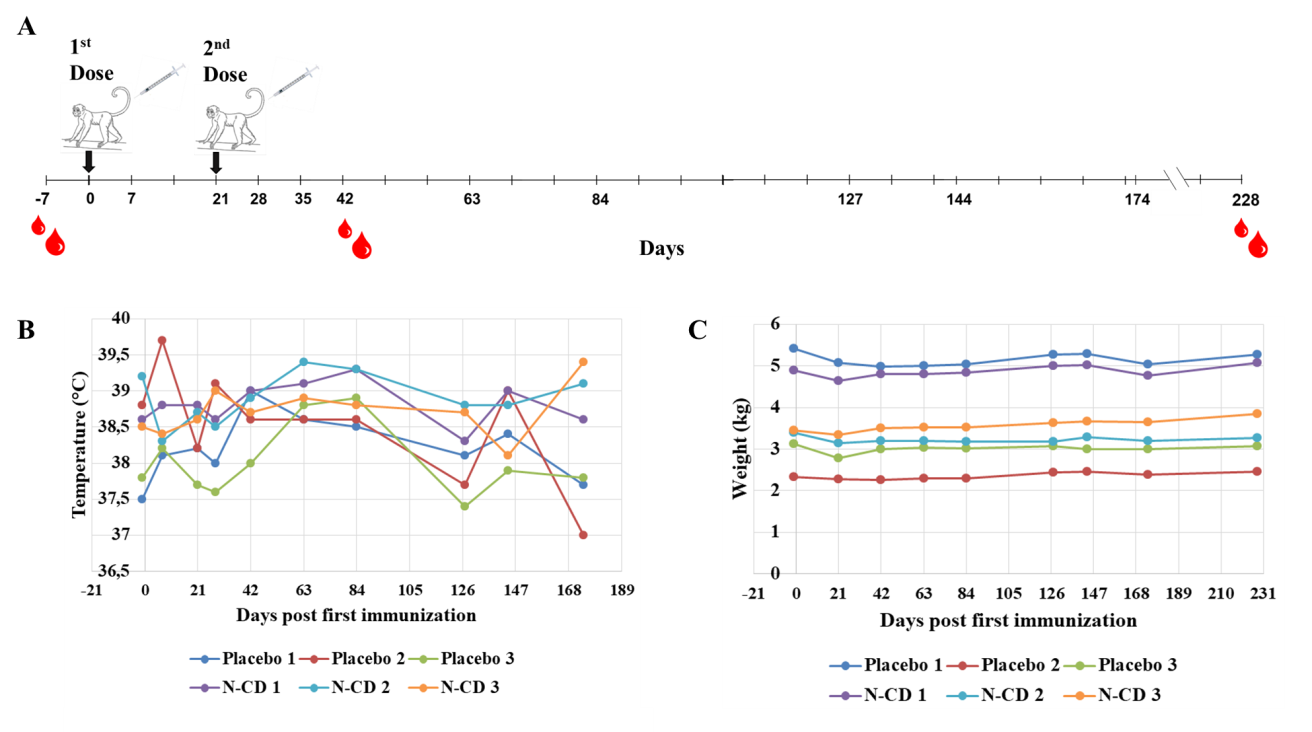


**Supplemental Fig. 4** Overall health monitoring of monkeys. **(A)** Animals were intramuscularly immunized with 50 µg of the N-CD protein or PBS (placebo) using alum as an adjuvant on days 0 and 21. Both experimental groups were composed by 3 animals. Blood draws were performed at -7, 42 and 228 days for hematological and biochemical tests. **(B)** and **(C)** Body temperature and weight, respectively, measured at different days during the experimentation schedule in monkeys.
