# Supplementary material for: Production and characterization of a chimeric antigen, based on nucleocapsid of SARS-CoV-2 fused to the extracellular domain of human CD154 in HEK-293 cells as a vaccine candidate against COVID-19
Source: PLoS One. 2023 Sep 26;18(9):e0288006. doi: 10.1371/journal.pone.0288006 (PMC10522030; doi:10.1371/journal.pone.0288006)
Supplement: S5 Fig — (A) Protein sequences of mouse, macaque, swine and human extracellular domain of CD154 were aligned using the bioinformatic tool Clustal Omega 2.1 (https:// www.ebi.ac.uk/Tools/msa/clustalo/). (B) Percent identity matrix of the aligned sequences. (*) Positions with a single, fully conserved residue. (:) Positions with conservation between amino acid groups of similar properties. (.) Positions with conservation between amino acid groups of weakly similar properties. (DOCX) [file pone.0288006.s005.docx]

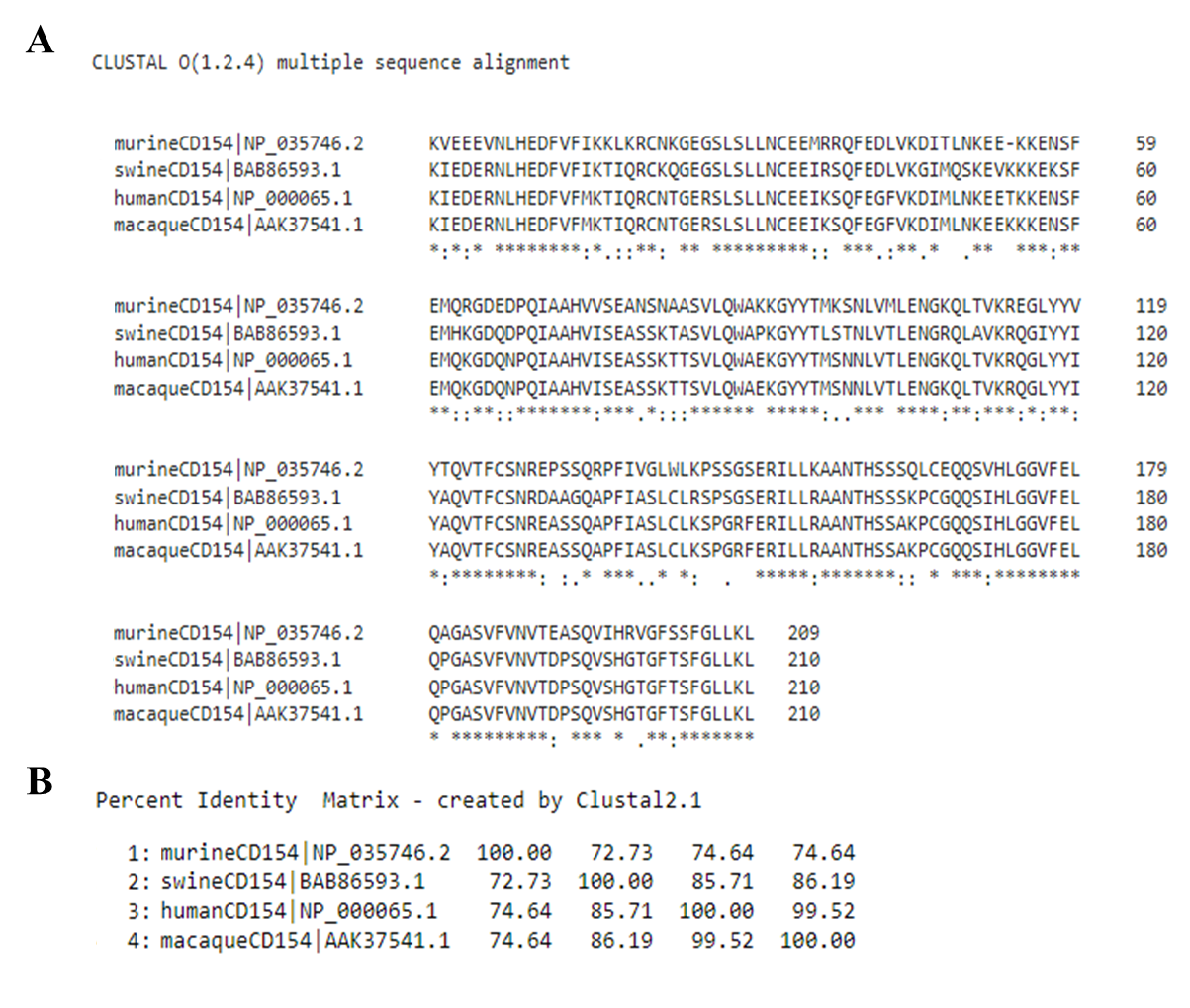
**Supplemental Fig. 5** Multiple sequence alignment of extracellular domain of CD154 from different species. **(A)** Protein sequences of mouse, macaque, swine and human extracellular domain of CD154 were aligned using the bioinformatic tool Clustal Omega 2.1 (https:// [www.ebi.ac.uk/Tools/msa/clustalo/](http://www.ebi.ac.uk/Tools/msa/clustalo/)). **(B)** Percent identity matrix of the aligned sequences. (*) Positions with a single, fully conserved residue. (:) Positions with conservation between amino acid groups of similar properties. (.) Positions with conservation between amino acid groups of weakly similar properties.
