# Supplementary material for: Production and characterization of a chimeric antigen, based on nucleocapsid of SARS-CoV-2 fused to the extracellular domain of human CD154 in HEK-293 cells as a vaccine candidate against COVID-19
Source: PLoS One. 2023 Sep 26;18(9):e0288006. doi: 10.1371/journal.pone.0288006 (PMC10522030; doi:10.1371/journal.pone.0288006)

Original film from western Figure 1B


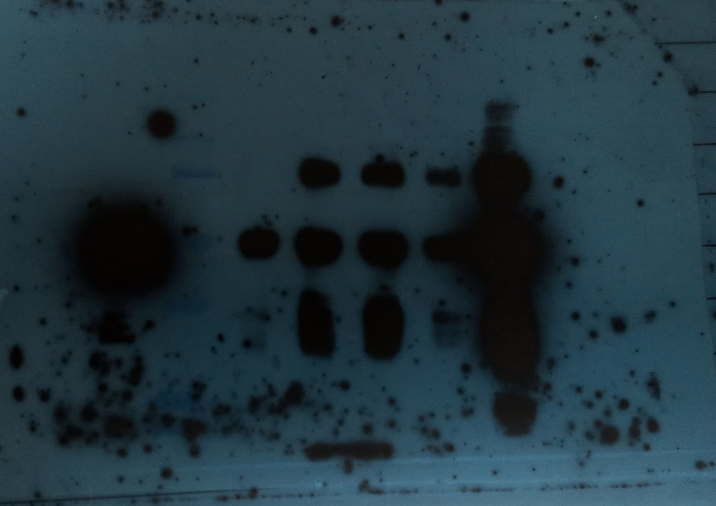


Original image for SDS-PAGE Figure 2B


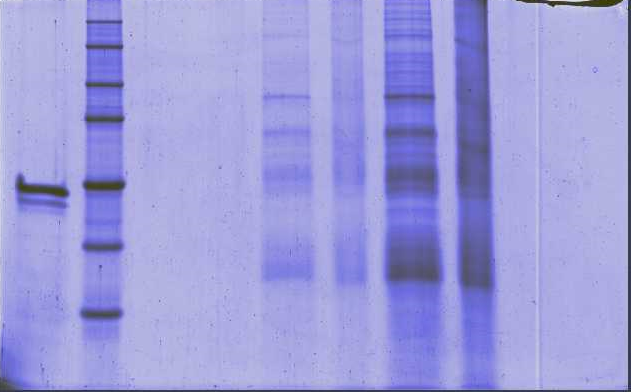


Original image for Western blot Figure 2C


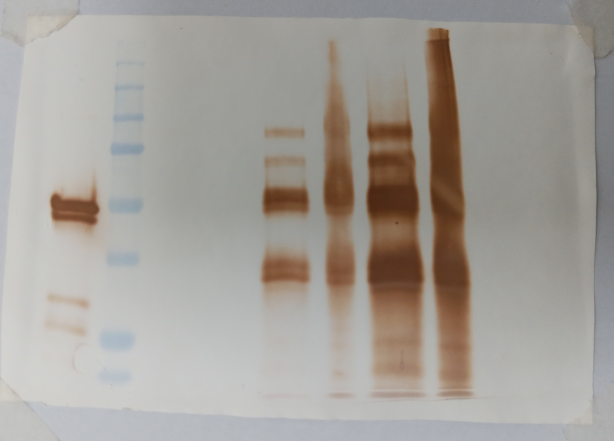


Original image from western blot Supplementary Figure 1C


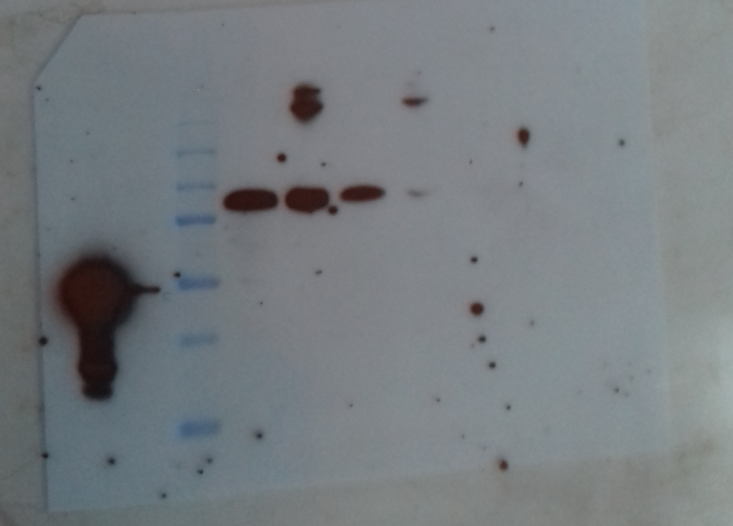


Original image from western blot Supplementary Figure 1D


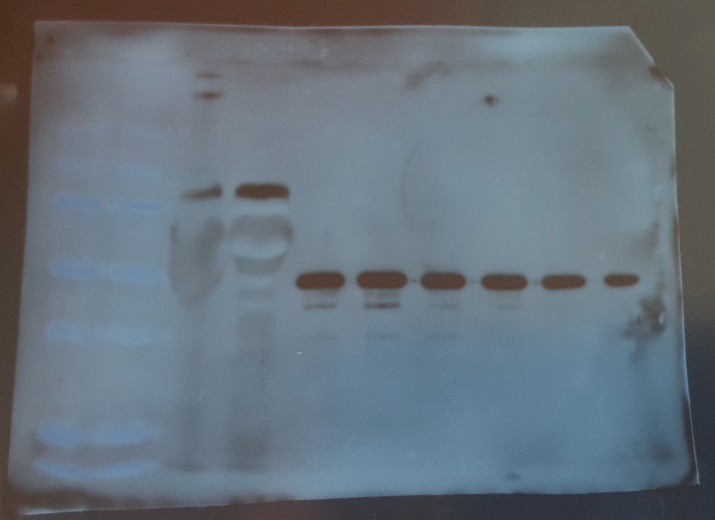

Supplement: S6 Fig — (DOCX) [file pone.0288006.s006.docx]
