# Supplementary material for: Production and characterization of a chimeric antigen, based on nucleocapsid of SARS-CoV-2 fused to the extracellular domain of human CD154 in HEK-293 cells as a vaccine candidate against COVID-19
Source: PLoS One. 2023 Sep 26;18(9):e0288006. doi: 10.1371/journal.pone.0288006 (PMC10522030; doi:10.1371/journal.pone.0288006)
Supplement: S1 Table — (DOCX) [file pone.0288006.s007.docx]

**Supplemental Table 1** List of oligonucleotides used for N gene isolation and cloning

| **Name** | **Sequence** |
| --- | --- |
| Primer 1.1 | 5’-GGGCATCATCACCATCACCATTCGAATTCTGGCGG-3’ |
| Primer 1.2 | 5’-GCCGCCGCCAGAATTCGAATGGTGATGGTGATGATGCCC-3’ |
| Primer 2.1 | 5’-CGGCTCCGGAGGGGGAGGGAGCGGCGGAGGGGGCTCCCCGC-3’ |
| Primer 2.2 | 5’-GGGGAGCCCCCTCCGCCGCTCCCTCCCCCTCCGGA-3’ |
| hCD154-F | 5´-ATATATCCGCGGAAGATAGAAGATGAAAGGAATC-3´ |
| hCD154-R | 5´-GTATATGTCGACCTAGAGCTTGAGTAAGCCAAAGG-3´ |
| N protein-F | 5´- TCTGATAATGGACCCCAAAATCAG-3´ |
| N protein-R | 5´- GGCCTGAGTTGAGTCAGCACTG-3´ |
| N-Display-F | 5´- ATATATGGATCCTCTGATAATGGACCCCAAAATC-3´ |
| N-Display-R | 5´- GGCCTGAGTTGAGTCAGCACTGCTC-3´ |
| N-pl6-F | 5´- ATATATCCCGGGGCGCGCGTTGACATTGATTATTG-3´ |
