# Supplementary material for: Production and characterization of a chimeric antigen, based on nucleocapsid of SARS-CoV-2 fused to the extracellular domain of human CD154 in HEK-293 cells as a vaccine candidate against COVID-19
Source: PLoS One. 2023 Sep 26;18(9):e0288006. doi: 10.1371/journal.pone.0288006 (PMC10522030; doi:10.1371/journal.pone.0288006)
Supplement: S2 Table — (DOCX) [file pone.0288006.s008.docx]

**Supplemental Table 2** Summary of the ESI–MS analysis for the in gel digestion protocols and sequence coverage of N-CD protein showed in Suppl. Fig. 2.

| **Fragment** | **Residue** | **Z** | **m/z Theor** | **Bands** | **Sequence** |
| --- | --- | --- | --- | --- | --- |
| T1 | 1-46 | 2+ | 899.41 | **1,2** | DYPYD VPDYA GAQPA R |
| T2 | 37-47 | 2+ | 600.27 | **3** | SPSDN GPQNQ R |
| T4 | 52-69 | 2+ | 912.41 | **1** | ITFGG PSDST GSNQN GER |
| T11 | 106-125 | 2+ | 1091.01 | **1,2,3** | GQGVP INTNS SPDDQ IGYYR |
| T19 | 145-164 | 2+ | 1134.04 | **1,2,3** | WYFYY LGTGP EAGLP YGANK |
| T20 | 165-180 | 2+ | 842.95 | **1,2,3** | DGIIW VATEG ALNTP K |
| T22 | 187-206 | 2+ | 1030.58 | **1,2,3** | NPANN AAIVL QLPQG TTLPK |
| T27 | 229-232 | 1+ | 463.23 | **2** | NSSR |
| T29 | 241-246 | 1+ | 588.31 | **2** | GTSPA R |
| T30 | 247-263 | 2+ | 835.95 | **1,2** | MAGNG GDAAL ALLLL DR (Partial deamination) |
| T45 | 357-375 | 2+ | 1013.02 | **1,2,3,4** | IGMEV TPSGT WLTYT GAIK |
| T46 | 376-379 | 1+ | 490.25 | **2,4** | LDDK |
| T50 | 399-406 | 2+ | 458.74 | **1,3,4** | TFPPT EPK |
| T59 | 426-442 | 2+ | 931.48 | **1,2,3,4** | QQTVT LLPAA DLDDF SK |
| T63 | 489-498 | 2+ | 640.31 | **1,2,3,4** | NLHED FVFMK |
| T66 | 509-519 | 2+ | 660.35 | **1,2,3,4** | SLSLL NCEEI K |
| T67 | 520-527 | 2+ | 471.24 | **1,2,3,4** | SQFEG FVK |
| T68 | 528-533 | 1+ | 733.39 | **2** | DIMLN K |
| T72 | 547-564 | 2+ | 926.46 | **1,2,3,4** | GDQNP QIAAH VISEA SSK |
|  |  | 3+ | 617.98 | **1,2,3,4** |  |
| T73 | 565-574 | 1+ | 1162.61 | **1,3,4** | TTSVL QWAEK* |
|  |  | 2+ | 581.81 | **1,2,3,4** |  |
| T74 | 575-590 | 2+ | 902.43 | **1,2,3,4** | GYYTM SNNLV TLENG K |
| T78 | 613-627 | 2+ | 818.42 | **1,2,3,4** | EASSQ APFIA SLCLK |
|  | | | | | |
| Assignments according to partial trypsin digestion | | | | | |
| T9-10 | 99-105 | 2+ | 452.75 | **1** | EDLKF PR |
| T25-26 | 223-228 | 1+ | 679.35 | **1,2,4** | SSSRS R* |
| T41-42 | 314-330 | 2+ | 972.98 | **1,2,3,4** | RGPEQ TQGNF GDQEL IR |
| FK-T48-T49 | 363-378 | 3+ | 649.04 | **1,2,3,4** | FKDQV ILLNK HIDAY K |
| R-T78-T79 | 592-611 | 4+ | 504.53 | **1,2,3,4** | REASS QAPFI ASLCacmL KSPGR |
|  |  | 3+ | 672.37 | **1,2,3,4** |  |

(*) Peptides verified by its mass but not by MS/MS. (Nprot-Red, CD154-Blue).
