# Supplementary material for: Production and characterization of a chimeric antigen, based on nucleocapsid of SARS-CoV-2 fused to the extracellular domain of human CD154 in HEK-293 cells as a vaccine candidate against COVID-19
Source: PLoS One. 2023 Sep 26;18(9):e0288006. doi: 10.1371/journal.pone.0288006 (PMC10522030; doi:10.1371/journal.pone.0288006)
Supplement: S3 Table — Nd (not determined). (DOCX) [file pone.0288006.s009.docx]

**Supplemental Table 3** Blood biochemical data for monkeys seven days before the first immunization. Nd (not determined).

| **Parameters** | **Abbreviation** | **Unit** | **Placebo-1** | **Placebo-2** | **Placebo-3** | **N-CD-1** | **N-CD-2** | **N-CD-3** |
| --- | --- | --- | --- | --- | --- | --- | --- | --- |
| Globulin index | A/G |  | 1,5 | 1,7 | 1,3 | 1,6 | 2,2 | 1,4 |
| Alanine amino transferase | ALAT | u/L | 8 | 14 | 4 | 5 | 2 | 5 |
| Aspartate amino transferase | ASAT | u/L | 37 | 58 | 53 | 49 | 24 | 46 |
| Alkaline phosphatase | ALP | u/L | 305 | 540 | 351 | 445 | 415 | 337 |
| Creatinine | CREA | µmol/L | 85 | 55 | 42 | 83 | 50 | 59 |
| Total proteins | TP | g/L | 80 | 70,2 | 79,9 | 74,7 | 68,3 | 75 |
| Albumin | ALB | g/L | 51,2 | 43,4 | 45,8 | 46,3 | 47,1 | 43,8 |
| Glucose | GLU | mmol/L | 6,55 | 4,89 | 3,31 | 8,97 | 4,82 | 4,1 |
| Cholesterol | CHOL | mmol/L | 4,41 | 3,54 | 3,88 | 3,35 | 2,53 | 3 |
| Total bilirubin | BIL-T | µmol/L | 0 | 0,2 | 0 | 0,4 | 0,3 | 0,2 |
| Direct bilirubin | BIL-D2 | µmol/L | 0,7 | 0,9 | 0 | 0,5 | 0,6 | 0,2 |
| Triglycerides | TG | mmol/L | 0,42 | 0,44 | 0,52 | 0,57 | 0,46 | 0,64 |
| Phosphorus | PHOS | mmol/L | 1,9 | 1,67 | 2,02 | 2,13 | 1,59 | 1,34 |
| Urea | UREA | mmol/L | 10,74 | 11,06 | 10,69 | 9,13 | 9,15 | 8,45 |
| Calcium | Ca | mmol/L | 2,56 | 2,49 | 2,42 | 2,7 | 2,52 | 2,43 |
| Uric acid | UA | µmol/L | 0 | 2 | 5 | 0 | 4 | 0 |
| Gamma glutamyl transferase | GGT | u/L | 80 | 132 | 69 | 116 | 79 | 86 |
| Hemoglobin | HB | g/dL | 14,6 | 13,5 | 11,8 | 13,7 | 12,6 | 13,5 |
| Hematocrit | HTC | % | 48,3 | 46,1 | 39,4 | 41,1 | 43,2 | 44,9 |
| Erythrocyte | ETO | 10^3^/mm^3^ | 1,91 | 7,06 | 6,6 | 7,81 | 6,64 | 7,44 |
| Platelet | PLAT | 10^3^/mm^3^ | 395 | 407 | Nd | 402 | 590 | 472 |
| Medium corpuscular volume | MCV | fL | 61 | 65 | 60 | 60 | 65 | 60 |
| Mean corpuscular hemoglobin | MCH | pg | 18,5 | 19,2 | 17,8 | 17,6 | 19 | 18,2 |
| Mean corspuscular hemoglobin concentration | MCHC | g/dL | 39,7 | 29,3 | 29,8 | 29,2 | 29,2 | 30,1 |
