# Supplementary material for: Production and characterization of a chimeric antigen, based on nucleocapsid of SARS-CoV-2 fused to the extracellular domain of human CD154 in HEK-293 cells as a vaccine candidate against COVID-19
Source: PLoS One. 2023 Sep 26;18(9):e0288006. doi: 10.1371/journal.pone.0288006 (PMC10522030; doi:10.1371/journal.pone.0288006)
Supplement: S4 Table — Nd (not determined). (DOCX) [file pone.0288006.s010.docx]

**Supplemental Table 4** Blood biochemical data for monkeys 42 days after the first immunization. Nd (not determined)

| **Parameters** | **Abbreviation** | **Unit** | **Placebo-1** | **Placebo-2** | **Placebo-3** | **N-CD-1** | **N-CD-2** | **N-CD-3** |
| --- | --- | --- | --- | --- | --- | --- | --- | --- |
| Globulin index | A/G |  | 1,5 | 1,4 | 1,6 | 1,5 | 1,7 | 1,2 |
| Alanine amino transferase | ALAT | u/L | 13 | nd | 13 | 25 | 22 | 9 |
| Aspartate amino transferase | ASAT | u/L | 40 | 34 | 43 | 43 | 31 | 40 |
| Alkaline phosphatase | ALP | u/L | 251 | 534 | 360 | 345 | 274 | 317 |
| Creatinine | CREA | µmol/L | 89 | 65 | 44 | 84 | 58 | 69 |
| Total proteins | TP | g/L | 82,9 | 70,9 | 76,7 | 78,1 | 76,6 | 80,3 |
| Albumin | ALB | g/L | 50,2 | 41,7 | 47,3 | 46,3 | 48,4 | 44,1 |
| Glucose | GLU | mmol/L | 6,21 | 4,34 | 3,84 | 5,17 | 6,22 | 5,24 |
| Cholesterol | CHOL | mmol/L | 4,16 | 3,35 | 4,2 | 4,33 | 3,38 | 3,81 |
| Total bilirubin | BIL-T | µmol/L | 2,4 | 2,5 | 1,6 | 2,6 | 2,8 | 3,5 |
| Direct bilirubin | BIL-D2 | µmol/L | 0,9 | 1,2 | 1 | 1 | 1,2 | 1,4 |
| Triglycerides | TG | mmol/L | 0,26 | 0,83 | 0,66 | 0,94 | 0,6 | 0,62 |
| Phosphorus | PHOS | mmol/L | 2 | 2,18 | 1,96 | 2,29 | 1,65 | 1,77 |
| Urea | UREA | mmol/L | 10,31 | 8,71 | 10,01 | 8,64 | 7,88 | 8,41 |
| Calcium | Ca | mmol/L | 2,6 | 2,32 | 2,43 | 2,67 | 2,68 | 2,4 |
| Uric acid | UA | µmol/L | 0 | 1 | 1 | 3 | 0 | 0 |
| Gamma glutamyl transferase | GGT | u/L | 90 | 111 | 68 | 96 | 75 | 81 |
| Hemoglobin | HB | g/dL | 13 | 13,2 | 11,2 | 14,3 | 14,4 | 14,3 |
| Hematocrit | HTC | % | 45,9 | 46,9 | 40,9 | 50,7 | 51,2 | 50,7 |
| Erythrocyte | ETO | 10^3^/mm^3^ | 6,34 | 6,14 | 5,89 | 7,03 | 6,57 | 6,95 |
| Platelet | PLAT | 10^3^/mm^3^ | 665 | 378 | 403 | Nd | 399 | 446 |
| Medium corpuscular volume | MCV | fL | 72 | 76 | 69 | 72 | 78 | 73 |
| Mean corpuscular hemoglobin | MCH | pg | 20,5 | 21,5 | 19,1 | 20,4 | 21,9 | 20,5 |
| Mean corspuscular hemoglobin concentration | MCHC | g/dL | 28,3 | 28,1 | 27,5 | 28,2 | 28,1 | 28,2 |
| Total leukocyte count | LEU | 10^3^/mm^3^ | 6,1 | 7,4 | 9,3 | 9,1 | Nd | 9,1 |
