# Supplementary material for: Production and characterization of a chimeric antigen, based on nucleocapsid of SARS-CoV-2 fused to the extracellular domain of human CD154 in HEK-293 cells as a vaccine candidate against COVID-19
Source: PLoS One. 2023 Sep 26;18(9):e0288006. doi: 10.1371/journal.pone.0288006 (PMC10522030; doi:10.1371/journal.pone.0288006)
Supplement: S5 Table — (DOCX) [file pone.0288006.s011.docx]

**Supplemental Table 5** Blood biochemical data for monkeys 228 days after the first immunization.

| **Parameters** | **Abbreviation** | **Unit** | **Placebo-1** | **Placebo-2** | **Placebo-3** | **N-CD-1** | **N-CD-2** | **N-CD-3** |
| --- | --- | --- | --- | --- | --- | --- | --- | --- |
| Globulin index | A/G |  | 2,2 | 1,8 | 2,3 | 2 | 2,6 | 1,8 |
| Alanine amino transferase | ALAT | u/L | 17,4 | 13 | 10,1 | 17,7 | 7,5 | 6,2 |
| Aspartate amino transferase | ASAT | u/L | 24,4 | 36,9 | 33,8 | 36,5 | 27,3 | 34,8 |
| Alkaline phosphatase | ALP | u/L | 144 | 385 | 294 | 243 | 255 | 237 |
| Creatinine | CREA | µmol/L | 82 | 74 | 49 | 68 | 65 | 83 |
| Total proteins | TP | g/L | 77 | 74 | 77 | 68 | 71 | 81 |
| Albumin | ALB | g/L | 53 | 48 | 54 | 45 | 51 | 52 |
| Glucose | GLU | mmol/L | 6,13 | 5,3 | 3,3 | 4,22 | 4,02 | 5,15 |
| Cholesterol | CHOL | mmol/L | 2,89 | 3,23 | 4,22 | 2,83 | 3,03 | 3,47 |
| Total bilirubin | BIL-T | µmol/L | 1,2 | 1,4 | 1,2 | 1,1 | 1 | 1,6 |
| Direct bilirubin | BIL-D2 | µmol/L | 0,7 | 0,6 | 0,6 | 0,6 | 0,7 | 0,9 |
| Triglycerides | TG | mmol/L | 0,5 | 0,71 | 0,62 | 0,7 | 0,96 | 1,14 |
| Phosphorus | PHOS | mmol/L | 1,71 | 1,71 | 2,02 | 1,65 | 1,33 | 0,86 |
| Urea | UREA | mmol/L | 13,5 | 11 | 14,3 | 9,2 | 11,6 | 13,6 |
| Calcium | Ca | mmol/L | 2,53 | 2,58 | 2,5 | 2,38 | 2,67 | 2,67 |
| Uric acid | UA | µmol/L | 1 | 1 | 1 | 3 | 1 | 1 |
| Gamma glutamyl transferase | GGT | u/L | 102 | 101 | 67 | 92 | 102 | 87 |
| Hemoglobin | HB | g/dL | 11,8 | 12,1 | 11,6 | 10,6 | 11,3 | 13,6 |
| Hematocrit | HTC | % | 41,2 | 43,3 | 40,8 | 38,1 | 39,7 | 48,7 |
| Erythrocyte | ETO | 10^3^/mm^3^ | 6,14 | 6,05 | 6,22 | 5,87 | 5,52 | 7,28 |
| Platelet | PLAT | 10^3^/mm^3^ | 373 | 285 | 428 | 361 | 448 | 452 |
| Medium corpuscular volume | MCV | fL | 67 | 72 | 66 | 65 | 72 | 67 |
| Mean corpuscular hemoglobin | MCH | pg | 19,2 | 20 | 18,6 | 18,1 | 20,4 | 18,7 |
| Mean corspuscular hemoglobin concentration | MCHC | g/dL | 28,5 | 27,9 | 28,4 | 27,9 | 28,4 | 27,9 |
| Total leukocyte count | LEU | 10^3^/mm^3^ | 7,4 | 9,8 | 10,7 | 6,2 | 10 | 9,9 |
| Neutrophils |  | % | 26 | 26 | 42 | 30 | 30 | 49 |
| Lymphocytes |  | % | 70 | 74 | 55 | 66 | 70 | 51 |
| Monocytes |  | % | 2 | 0 | 2 | 1 | 0 | 0 |
| Eosinophils |  | % | 2 | 0 | 1 | 3 | 0 | 0 |
| Basophils |  | % | 0 | 0 | 0 | 0 | 0 | 0 |
