# Supplementary material for: Production and characterization of a chimeric antigen, based on nucleocapsid of SARS-CoV-2 fused to the extracellular domain of human CD154 in HEK-293 cells as a vaccine candidate against COVID-19
Source: PLoS One. 2023 Sep 26;18(9):e0288006. doi: 10.1371/journal.pone.0288006 (PMC10522030; doi:10.1371/journal.pone.0288006)
Supplement: S6 Table — The absorbance values obtained from the assessment of the sera from monkeys immunized with the N-CD protein or the placebo group, using a peptide from SARS-CoV-2-N protein or the complete protein as coating antigen is showed. The absorbance values of the assay controls such as positive, negative and blank controls are also depicted in this table. A positive result was considered when the mean absorbance values were higher than 0.18 (media plus three times the standard deviation of absorbance values of negative control). Positive control: Serum from convalescent COVID-19 patient. Negative control: Serum from non-infected patient. Blank control: wells were filled only with washing buffer. (DOCX) [file pone.0288006.s012.docx]

**Supplemental Table 6 Epitope mapping of antibodies against SARS-CoV-2 N protein by ELISA.** The absorbance values obtained from the assessment of the sera from monkeys immunized with the N-CD protein or the placebo group, using a peptide from SARS-CoV-2-N protein or the complete protein as coating antigen is showed. The absorbance values of the assay controls such as positive, negative and blank controls are also depicted in this table. A positive result was considered when the mean absorbance values were higher than 0.18 (media plus three times the standard deviation of absorbance values of negative control). Positive control: Serum from convalescent COVID-19 patient. Negative control: Serum from non-infected patient. Blank control: wells were filled only with washing buffer.
